# Gold(I) Complexes of 9-Deazahypoxanthine as Selective Antitumor and Anti-Inflammatory Agents

**DOI:** 10.1371/journal.pone.0109901

**Published:** 2014-10-15

**Authors:** Ján Vančo, Jana Gáliková, Jan Hošek, Zdeněk Dvořák, Lenka Paráková, Zdeněk Trávníček

**Affiliations:** 1 Regional Centre of Advanced Technologies and Materials & Department of Inorganic Chemistry, Faculty of Science, Palacký University, Olomouc, Czech Republic; 2 Regional Centre of Advanced Technologies and Materials & Department of Cell Biology and Genetics, Faculty of Science, Palacký University, Olomouc, Czech Republic; 3 Department of Human Pharmacology and Toxicology, Faculty of Pharmacy, University of Veterinary and Pharmaceutical Sciences Brno, Brno, Czech Republic; Universidade Nova de Lisboa, Portugal

## Abstract

The gold(I) mixed-ligand complexes involving *O*-substituted derivatives of 9-deazahypoxanthine (HL*_n_*) and triphenylphosphine (PPh_3_) with the general formula [Au(L*_n_*)(PPh_3_)] (**1**–**5**) were prepared and thoroughly characterized by elemental analysis, FT-IR and multinuclear NMR spectroscopy, ESI+ mass spectrometry, single crystal X-ray (HL_5_ and complex **2**) and TG/DTA analyses. Complexes **1**–**5** were evaluated for their *in vitro* antitumor activity against nine human cancer lines, i.e. MCF7 (breast carcinoma), HOS (osteosarcoma), A549 (adenocarcinoma), G361 (melanoma), HeLa (cervical cancer), A2780 (ovarian carcinoma), A2780R (ovarian carcinoma resistant to *cisplatin*), 22Rv1 (prostate cancer) and THP-1 (monocytic leukaemia), for their *in vitro* anti-inflammatory activity using a model of LPS-activated macrophages, and for their *in vivo* antiedematous activity by λ-carrageenan-induced hind paw edema model on rats. The results showed that the complexes **1**–**5** exhibit selective *in vitro* cytotoxicity against MCF7, HOS, 22Rv1, A2780 and A2780R, with submicromolar IC_50_ values for **2** against the MCF7 (0.6 µM) and HOS (0.9 µM). The results of in vitro cytotoxicity screening on primary culture of human hepatocytes (HEP220) revealed up to 30-times lower toxicity of compounds against healthy cells as compared with cancer cells. Additionally, the complexes **1**–**5** significantly influence the secretion and expression of pro-inflammatory cytokines TNF-α and IL-1β by a similar manner as a commercially used anti-arthritic drug Auranofin. The tested complexes also significantly influence the rate and overall volume of the edema, caused by the intraplantar application of λ-carrageenan polysaccharide to rats. Based on these promising results, the presented compounds could qualify to become feasible candidates for advanced testing as potential antitumor and anti-inflammatory drug-like compounds.

## Introduction

**Figure 1:**
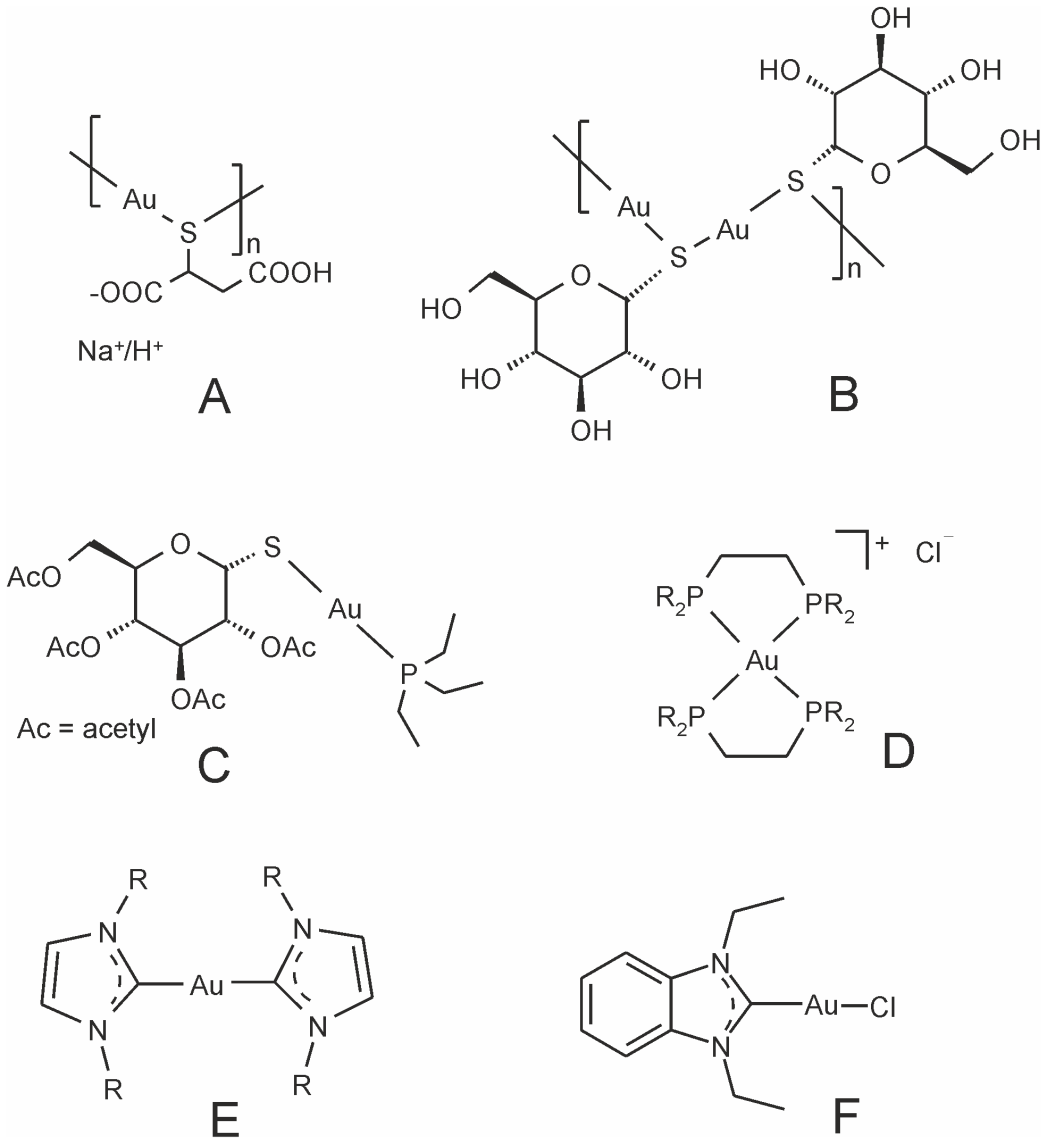
Schematic representations of gold-containing anti-inflammatory drugs (A–C) and some anticancer drug candidates (D–F).

Medicinal use of gold-based therapeutic agents can be traced back to 2500 BC in China [1], [2]. Currently, the foremost clinical use of gold compounds is related to their application in the treatment of rheumatoid arthritis. The most important clinically used gold-based anti-arthritic drugs are various gold(I) thiolate salts, e.g. sodium aurothiomalate (Myochrysin, sodium ((2-carboxy-1-carboxylatoethyl)thiolato)gold(I)), Figure 1A) and aurothioglucose (Solganol, {(2*S*,3*R*,4*S*,5*S*,6*R*)-3,4,5-trihydroxy-6-(hydroxymethyl)-oxane-2-thiolato}gold(I), Figure 1B) [3], [4] belonging to the class of disease-modifying anti-rheumatic drugs so-called DMARDs, and an orally active gold(I) phosphine compound Auranofin (Ridaura, triethylphosphine-(2,3,4,6-tetra-*O*-acetyl-β-D-thiopyranosato)gold(I), Figure 1C) [5]–[7]. Over the past few years, research interests in medicinal chemistry of gold compounds have not been focused only on the development of gold-based drugs with better or comparable efficiency, and/or fewer negative side-effects than commercially clinically used anti-rheumatoid agents, but also on the study of the mode of action of gold compounds in the physiological environment with the aim to understand the relationship between the mechanism and anti-inflammatory activity as well as possible variety of their biological applications, e.g. anti-cancer, anti-microbial, anti-malarial and anti-HIV activities [8]–[15].

Deeper investigations of gold-based compounds as potential antitumor agents started when commercially used anti-arthritic drugs such as Auranofin and gold(I) thiolate salts showed promising results of cell growth inhibiting effects *in vitro* [16]–[18] and some efficacy in experimental *in vivo* models [19]–[20]. Accordingly, numerous Auranofin analogues, i.e. linear Au(I) phosphine complexes incorporating *S*-donor ligands [21]–[26] or heterocyclic *N*-donor ligands [27]–[30] as well as various analogues of tetrahedral gold(I) diphosphines e.g. of the type [Au(DPPE)_2_]Cl, where DPPE represents a tetrasubstituted ethylene-1,2-diphosphine ligand [31]–[35] (for a representative example see Figure 1D) and Au(I) *N*-heterocyclic carbene (NHC) complexes of the type [Au(NHC)_2_] and [Au(NHC)Cl], where NHC is a heterocyclic carbene ligand derived from *N*,*N*'-disubstituted imidazole [36]–[37] (for representative examples see Figure 1E and 1F), have gained more attention due to their cytotoxicity and/or antitumor activity against several tumor cell lines/models, e.g. melanoma and leukaemia cell lines/leukaemia model.

Not surprisingly, the studies focusing on the class of gold(I) compounds containing triphenylphosphine and heterocyclic *N*-donor ligands (L), with the general composition [Au(L)(PPh_3_)], described both anti-inflammatory and cytotoxic activities and also showed clinical potential of these compounds in the treatment of anti-inflammatory diseases or cancer. In relation, significant *in vitro* cytotoxicity against breast MCF7, lung A549, cervical (A431) colon (LoVo cell line and multi-drug resistant LoVo MDR cell line), ovarian (2008 and C13*) cancers was described for gold(I) complexes with triphenylphosphine and imidazoles [27]. Further, gold(I) complexes involving triphenylphosphine and 6-benzyladenine (HBap) derivatives, [Au(Bap)(PPh_3_)], showed better anti-inflammatory effect and lower *in vitro* cytotoxicity as compared with the commercially used drug Auranofin [38].

9-Deazahypoxanthine derivatives (6-oxo-9-deazapurines) as inhibitors of purine nucleoside phoshorylase (PNP) [39], represent a novel class of prospective selective immunosuppressive agents with potential utilization in the treatment of autoimmune and T-cell proliferative diseases such as T-cell leukaemia and lymphomas [40]. The immunicillin family (C9-substituted 9-deazahypoxanthines) represents the most powerful PNP inhibitors, with two members, immucillin-H and DADMe-immucillin-H, included in clinical trials for the treatment of T-cell and B-cell cancers [41]–[42].

One of the possible ways how to obtain metal-based drugs with improved biological activity is a coordination of suitable ligands to a proper metal. This general pathway, which is associated with a combination of suitable ligands with a proper transition metal (all these components being partly biologically active or even biologically inactive), may lead to the formation of biologically active compounds [e.g. 38, 43–45]. Despite the above-mentioned biological potential of 9-deazahypoxanthine derivatives in clinical applications, its coordination chemistry is still quite new. To date, only one work has been published in connection with the study of coordination compounds incorporating the molecule of 9-deazahypoxanthine [46].

In this work, we wish to present the preparation, characterization and evaluation of *in vitro* and *in vivo* antitumor and anti-inflammatory activities of a series of gold(I)-triphenylphosphine complexes with the general formula [Au(L*_n_*)(PPh)_3_], where HL*_n_* represents an *O*-substituted 9-deazahypoxanthine derivative.

As a model of *in vitro* inflammatory response, the expression of pro-inflammatory cytokine tumor necrosis factor α (TNF-α) and interleukin 1β (IL-1β) were determined in lipopolysaccharide (LPS)-stimulated macrophage-like cells THP-1. TNF-α plays an important role during inflammation, as it stimulates the expression of other cytokines and adhesion molecules, causes vasodilatation, participates on generation of reactive oxygen species (ROS), and also possesses other effects on inflamed tissues [47]. The IL-1β cytokine represents another key molecule involved in inflammation processes. It influences signalling pathways leading to fever, expression and activation of other inflammatory-related agents [48]–[49]. Both of these cytokines are under transcription control of the nuclear factor κB (NF-κB) since the LPS activation of NF-κB signalling pathway leads to the cleavage of its complex with the inhibitor of NF-κB (IκB), while the free NF-κB is translocated from cytoplasm to the nucleus and initiates the transcription of several hundreds of genes, including the TNF-α and IL-1β [50].

Overall positive results and findings related to biological activities are discussed within the framework of the following text.

## Materials and Methods

### Ethic Statement

This study was carried out in strict accordance with the recommendations in the Guide for the Care and Use of Laboratory Animals of the National Institute of Health [51]. The protocol was approved by the Expert Committee on the Protection of Animals Against Cruelty at the University of Veterinary and Pharmaceuticals Science in Brno (Permit Number: 73-2013). To minimize the suffering of laboratory animals, all pharmacological interventions were done under anaesthesia. The animal tissues for *ex vivo* experiments were taken *post mortem*, immediately after all animals were sacrificed by cervical dislocation.

### Chemicals and Biochemicals

Chemicals and solvents used for the synthesis of *O*-substituted 9-deazahypoxanthine derivatives, HL*_n_* (*n* = 1–5, HL_1_ = 6-ethoxy-9-deazapurine, HL_2_ = 6-isopropyloxy-9-deazapurine, HL_3_ = 6-(tetrahydrofuran-2-yl-methyloxy)-9-deazapurine, HL_4_ = 6-benzyloxy-9-deazapurine, HL_5_ = 6-phenethyloxy-9-deazapurine), and gold(I) triphenylphosphine complexes **1**–**5** were purchased from Across Organics Co. (Pardubice, Czech Republic), Sigma-Aldrich Co. (Prague, Czech Republic) and Fisher-Scientific Co. (Pardubice, Czech Republic), and were used without any further purification. The precursor [AuCl(PPh_3_)] was prepared by the synthetic procedure described in the literature [52]–[53].

The RPMI 1640 medium and penicillin-streptomycin mixture were purchased from Lonza (Verviers, Belgium). Phosphate-buffered saline (PBS), fetal bovine serum (FBS), phorbol myristate acetate (PMA), prednisone (≥98%), Auranofin (≥98%), erythrosin B, and *Escherichia coli* 0111∶B4 lipopolysaccharide (LPS) were purchased from Sigma-Aldrich (Steinheim, Germany). Cell Proliferation Reagent WST-1, Cell Proliferation Kit I (MTT), cOmplete Proteinase Inhibitor Cocktail, and RealTime Ready Cell Lysis Kit used for the isolation of RNA from cells, and Transcriptor Universal cDNA Master used for reverse transcription of RNA to cDNA were obtained from Roche (Mannheim, Germany). Specific primers and probes (Gene Expression assays) for polymerase chain reaction (PCR) were obtained from Applied Biosystems (Foster City, CA, USA). The following assays were chosen for the quantification of gene expression: Hs00174128_m1 for TNF-α, Hs01555410_m1 for IL-1β, and 4326315E for β-actin, which served as an internal control of the gene expression. Quantitative PCR (qPCR) was performed with Fast Start Universal Probe Master from Roche (Mannheim, Germany). Instant ELISA Kits from eBioscience (Vienna, Austria) were used to evaluate the production of TNF-α and IL-1β by the enzyme linked immunosorbent assay (ELISA) method. The Immun-Blot PVDF (polyvinylidene fluoride) membrane 0.2 µm from Bio-Rad (Hercules, CA, USA) and albumin bovine fraction V (pH 7) (BSA) from Serva (Heidelberg, Germany) were used for Westernblot. Murine monoclonal anti-IκB-α from Cell Signaling (Danvers, MA, USA), murine monoclonal anti-β-actin from Abcam (Cambridge, UK) and goat polyclonal anti-mouse IgG (with conjugated peroxidase) antibodies from Sigma-Aldrich (Saint Louis, MO, USA) were applied for immunodetection. Conjugated peroxidase was detected by Opti-4CN Substrate Kit from Bio-Rad (Hercules, CA, USA).

### Chemistry

The *O*-substituted 9-deazahypoxanthine derivatives, HL*_n_* (*n* = 1–5, HL_1_ = 6-ethoxy-9-deazapurine, HL_2_ = 6-isopropyloxy-9-deazapurine, HL_3_ = 6-(tetrahydrofuran-2-yl-methyloxy)-9-deazapurine, HL_4_ = 6-benzyloxy-9-deazapurine, HL_5_ = 6-phenethyloxy-9-deazapurine) were synthesized by a slight modification of the procedure involving the nucleophilic substitutions as published previously [54]–[55]. The purity and composition of the products were confirmed by elemental analysis (C, H, N), electrospray ionization (ESI+) mass spectrometry, FT-IR, ^1^H and ^13^C NMR spectroscopies, results of which are given in Information S1, including the detailed synthetic procedure of HL_1–5_. The molecular structure of HL_5_ was determined by single crystal X-ray analysis (for further details see Information S1).

Gold(I) complexes of the composition [Au(L_1–5_)(PPh_3_)] (**1**–**5**), where L_1–5_ stands for the deprotonated form of the appropriate *O*-substituted 9-deazahypoxanthine derivative, were synthesized by a slightly modified procedure, as previously described in [38]. Accordingly, the acetone solutions of the appropriate *O*-substituted 9-deazahypoxanthine derivative (HL_1–5_) (0.2 mmol in 10 mL) and [AuCl(PPh_3_)] (0.2 mmol in 10 mL) were mixed. Then, an aqueous solution of 1 M NaOH (1 mL) was added and the reaction mixture was heated up to 50°C. The insoluble crystals of NaCl, formed during 2 hours of stirring, were filtered off. The colourless filtrate was evaporated to dryness by standing at room temperature. After a few days, the products **1**–**5** were precipitated by diethyl ether from the residue of gel-like consistency. The pale yellow powders were filtered off, washed with diethyl ether (5 mL) and dried at 40°C under an infrared lamp. The results of elemental analysis, ESI+ mass spectrometry, FT-IR spectroscopy, thermogravimetric (TG) and differential thermal (DTA) analyses are given in Information S1, including the selected crystallographic data and structure refinement of complex **2**.

### Physical Measurements

Elemental analyses (C, H, N) were carried out using a Flash 2000 CHNO-S Analyzer (Thermo Scientific, USA). FT-IR spectra were measured on a Nexus 670 spectrometer (Thermo Nicolet, USA) in the 400–4000 cm^−1^ (ATR technique) and 150–600 cm^−1^ (Nujol technique) regions. Mass spectra of the methanol solutions (*ca* 10^−5^ *M*) of complexes **1**–**5** were obtained by an LCQ Fleet ion trap mass spectrometer by the positive mode electrospray ionization (ESI+) technique (Thermo Scientific, USA). All the observed isotopic distribution representations were compared with the theoretical ones (QualBrowser software, version 2.0.7, Thermo Fischer Scientific). Simultaneous TG/DTA analyses were performed using an Exstar TG/DTA 6200 thermal analyzer (Seiko Instruments Inc., Japan); ceramic crucible, 150 mL min^−1^ dynamic air atmosphere, 25–850°C temperature range and temperature gradient of 2.5°C min^−1^. ^1^H and ^13^C NMR spectra and two dimensional correlation experiments (^1^H–^1^H gs-COSY, ^1^H–^13^C gs-HMQC, ^1^H–^13^C gs-HMBC; gs  =  gradient selected, COSY  =  correlation spectroscopy, HMQC  =  heteronuclear multiple quantum coherence, HMBC  =  heteronuclear multiple bond coherence) of the DMF-*d_7_* solutions were measured at 300 K on a Varian 400 device at 400.00 MHz (^1^H) and 100.58 MHz (^13^C). ^1^H and ^13^C spectra were adjusted against the signals of tetramethylsilane (Me_4_Si). The splitting of proton resonances in the reported ^1^H spectra is defined as s  =  singlet, d  =  doublet, t  =  triplet, br  =  broad band, dd  =  doublet of doublets, m  =  multiplet. The single crystal X-ray data of 6-phenethyloxy-9-deazapurine (HL_5_) and [Au(L_2_)(PPh_3_)] (**2**) were collected on a Xcalibur2 diffractometer (Oxford Diffraction Ltd., UK) equipped with a Sapphire2 CCD detector using the MoKα radiation (monochromator Enhance, Oxford Diffraction Ltd.), and ω-scan technique at 120K. Data collection, data reduction and cell parameter refinements were performed by the *CrysAlis* software package [56]. The molecular structures were solved by direct methods and all non-hydrogen atoms were refined anisotropically on *F^2^* with the full-matrix least-squares procedure (*SHELX-97*) [57]. H-atoms were located in difference maps and refined using the riding model. Molecular graphics were drawn and additional structural parameters were interpreted using *DIAMOND* [58].

### Maintenance and Preparation of Macrophages

For the determination of biological activity, we used the human monocytic leukaemia cell line THP-1 (ECACC, Salisbury, UK). The cells were cultivated at 37°C in the RPMI 1640 medium supplemented with 2 mM l-glutamine, 10% FBS, 100 U/mL of penicillin and 100 µg/mL of streptomycin in a humidified atmosphere containing 5% CO_2_. Stabilized cells (3^rd^–15^th^ passage) were split into microtitration plates to get a concentration of 500 000 cells/mL and the differentiation to macrophages was induced by phorbol myristate acetate (PMA) dissolved in dimethyl sulfoxide (DMSO) at the final concentration of 50 ng/ml, and the cells were incubated for 24 h. In comparison with monocytes, differentiated macrophages tend to adhere to the bottoms of the cultivation plates. For next 24 h the cells were incubated with a fresh complete RPMI medium, i.e. containing antibiotics and FBS, without PMA. The medium was then aspirated, and the cells were washed with PBS and cultivated for next 24 hours in serum-free RPMI 1640 medium. These prepared macrophages were used for the detection of inflammatory response.

### *In Vitro* Cytotoxicity Assay

*In vitro* cytotoxic activity was determined by the MTT assay in human breast adenocarcinoma (MCF7; ECACC no. 86012803), human osteosarcoma (HOS; ECACC no. 87070202), lung carcinoma (A549; ECACC no. 86012804), malignant melanoma (G361; ECACC no. 88030401), cervix epitheloid carcinoma (HeLa; ECACC no. 93021013), ovarian carcinoma (A2780; ECACC no. 93112519), *cisplatin*-resistant ovarian carcinoma (A2780R; ECACC no. 93112517) and prostate carcinoma (22Rv1; ECACC no 105092802) cancer cell lines, purchased from European Collection of Cell Cultures (ECACC). The cells were cultured according to the ECACC instructions and they were maintained at 37°C and 5% CO_2_ in a humidified incubator. The primary culture of human hepatocytes (HEP220, batch number HEP220819) was obtained from Biopredic International (France). The culture medium was Williams and HAM's F-12 (1∶1) supplemented with penicillin, streptomycin, ascorbic acid, linoleic acid, holo-transferin, ethanolamine, glucagon, insulin, dexamethasone, pyruvate, glucose, glutamine, amphotericin. The medium was enriched for plating with 2% foetal calf serum (v/v). The medium was exchanged for a serum-free medium the day after and the culture was stabilized for additional 24 h. Thereafter, the cells were ready for treatments. The cultures were maintained at 37°C and 5% CO_2_ in a humidified incubator. The cells were treated with complexes **1**–**5** (at the concentration levels of 0.01, 0.1, 1.0, 5.0, 25.0, and 50.0 µM), starting compounds e.g. HL_1–5_, AuCl, and *cisplatin* (applied up to 50 µM) for 24 h, using multi-well culture plates of 96 wells. In parallel, the cells were treated with vehicle (DMF; 0.1%, v/v) and Triton X-100 (1%, v/v) to assess the minimal (i.e. the positive control), and maximal (i.e. the negative control) cell damage, respectively. The MTT assay [59] was performed spectrophotometrically at 540 nm (TECAN, Schoeller Instruments LLC).

Before the *in vitro* anti-inflammatory testing, *in vitro* cytotoxicity on human monocytic leukaemia cells (THP-1, ECACC no. 88081201) was determined using the WST-1 assay. The THP-1 cells (floating monocytes, 500 000 cells/mL) were incubated in 100 µL of the serum-free RPMI 1640 medium and seeded into 96-well plates in triplicate at 37°C. Measurements were taken 24 h after the treatment with the tested compounds dissolved in 0.1% DMSO in the concentration range of 0.16–10.00 µM. Viability was determined by the WST-1 test according to the manufacturer's manual. The amount of the formed formazan (which correlates to the number of metabolically active cells in the culture) was calculated as a percentage of the control cells, which were treated only with 0.1% DMSO and was set-up as 100%. The IC_50_ values of the tested compounds were calculated from the obtained data. The WST-1 assay was performed spectrophotometrically at 440 nm (FLUOstar Omega, BMG Labtech).

### Drug Treatment and Induction of Inflammatory Response

Differentiated macrophages were pretreated with 300 nM solutions of the tested complexes, HL*_n_*, AuCl, [AuCl(PPh_3_)], PPh_3_ and Auranofin dissolved in DMSO (the final DMSO concentration was 0.1%) and with 0.1% DMSO solution itself (*vehicle*) for 1 h; the given concentrations of the tested compounds lack the cytotoxic effect (cell viability >94%) based on the results of WST-1 test. The inflammatory response in pretreated macrophages was triggered by the addition of 1.0 µg/mL lipopolysaccharide (LPS) dissolved in water, while the control cells (CTRL) remained without the LPS treatment.

### RNA Isolation and Gene Expression Evaluation

In the order to evaluate the expression of TNF-α, IL-1β, and β-actin mRNA, total RNA was isolated directly from the LPS-stimulated THP-1 cells. THP-1 macrophages were pretreated with compounds **2**, **5**, and Auranofin at the concentration of 300 nM or the vehicle (0.1% DMSO) only. After 1 h of the incubation, the inflammatory response was induced by LPS [except for the control cells (CTRL)]. After 2 h of the incubation with LPS, the medium was aspirated and the total RNA was isolated directly from the cells in cultivation plates using a RealTime Ready Cell Lysis Kit (Roche), according to the manufacturer's instructions.

The gene expression was quantified by two-step reverse-transcription quantitative (real-time) PCR (RT-qPCR). The reverse transcription step was performed by Transcriptor Universal cDNA Master using cell lysate as a template. The reaction consists of 3 steps: (1) primer annealing 29°C for 10 min; (2) reverse transcription 55°C for 10 min; and (3) transcriptase inactivation 85°C for 5 min. A FastStart Universal Probe Master and Gene Expression assays were used for qPCR. These assays contain specific primers and TaqMan probes that bind to an exon-exon junction to avoid DNA contamination. The parameters for the qPCR work were adjusted according to the manufacturer's recommendations: 50°C for 2 min, then 95°C for 10 min, followed by 40 cycles at 95°C for 15 s and 60°C for 1 min. The results were normalized to the amount of ROX reference dye, and the change in gene expression was determined by the 2^−ΔΔCT^ method [60]. A degree of transcription in the control cells (i.e. in the cells which were pretreated by vehicle only and not LPS stimulated) was set as 1 and other experimental groups were multiples of this value.

### Evaluation of Cytokine Secretion

Macrophages, which were pretreated with the tested compounds (complexes **1**–**5**, HL*_n_*, AuCl, [AuCl(PPh_3_)], PPh_3_ and Auranofin) for 1 h, were incubated with LPS for next 24 h. After this period, the medium was collected and the concentration of TNF-α, and IL-1β was determined by the Instant ELISA Kit according to the manufactures' manual.

### Determination of IκB degradation

Macrophage-like THP-1 cells were pretreated with the tested compounds and stimulated by LPS as was describe above. Thirty minutes after the addition of LPS, the medium was aspirated and cells were washed by cold phosphate buffer solution (PBS). Subsequently, the cells were collected using the lysis buffer [50 mM Tris-HCl pH 7.5, 1 mM EGTA, 1 mM EDTA, 1 mM sodium orthovanadate, 50 mM sodium fluoride, 5 mM sodium pyrophosphate, 270 mM sucrose, 0.1% (v/v) Triton X-100, and cOmplete Protease Inhibitor Cocktail (Roche, Germany)] and scraper. The lysis of cells was facilitated by a short (≈30 s) incubation in the ultrasonic water bath. The protein concentration was determined according to Bradford's method. For protein separation, 30 µg of protein was loaded onto the 12% polyacrylamide gel. Then, they were electrophoretically transferred on the PVDF membranes, which were subsequently blocked by 5% BSA dissolved in TBST buffer [150 mM NaCl, 10 mM Tris base pH 7.5, 0.1% (v/v) Tween-20]. The membranes were incubated with the primary anti-IκB-α antibody at the concentration ratio of 1∶500, or with the primary anti-β-actin at the concentration ration of 1∶5 000 at 4°C for 16 h. After washing, the secondary anti-mouse IgG antibody diluted 1∶2 000 was applied on the membranes and incubated at laboratory temperature (22°C) for 1 h. The amount of the bound secondary antibody was detected colorimetrically by an Opti-4CN Kit according to the manufacturer's manual.

### Animals

Wistar-SPF (6–8 weeks male) rats were obtained from AnLab, Ltd. (Prague, Czech Republic). The animals were kept in plexiglass cages at the constant temperature of 22±1°C and relative humidity of 55±5% for at least 1 week before the experiment. They were given food and water *ad libitum*. All the experimental procedures were performed according to the National Institutes of Health (NIH) Guide for the Care and Use of Laboratory Animals [51]. In addition, all the tests were conducted under the guidelines of the International Association for the Study of Pain [61]. After a one-week adaptation period, male Wistar-SPF rats (200–250 g) were randomly assigned to five groups (n = 7) of the animals in the study. The first, control group, received 25% DMSO (v/v in water, intraperitoneal; *i.p.*). The next three groups were pretreated with complexes **2**, **4** and **5**, and involved into the carrageenan-treatment. The fifth group was treated with a non-steroidal anti-inflammatory drug Indomethacin (5 mg/kg), which served as a positive control group (Indomethacin + carrageenan).

### Carrageenan-Induced Hind Paw Edema

The carrageenan-induced hind paw edema model was used for the determination of anti-inflammatory activity [62]. Animals were *i.p.* pretreated with complexes **2**, **4**, and **5** (the dosages of the individual complexes were adjusted to contain the same amount of gold as in 10 mg/kg dose of Auranofin), Indomethacin (5 mg/kg), or 25% DMSO (v/v in water) 30 min prior to the injection of 1% λ-carrageenan solution (50 µL) into the plantar side of right hind paws of the rats. The paw volume was measured immediately after the carrageenan injection (this value was set-up as baseline value for the hind paw volume) and during the next 6 h after the administration of the edematogenic agent using a plethysmometer (model 7159, Ugo Basile, Varese, Italy). The degree of swelling was evaluated as a percentage of the change of the volume of the right hind paw after the carrageenan treatment from the baseline volume. A non-steroidal anti-inflammatory drug Indomethacin was used as a positive control and the obtained data were also compared with the previously reported profile of antiedematous activity for Auranofin [38]. After 6 h, the animals were sacrificed and the edematous feet were dissected, the tissue from the plantar parts was extracted, fixed and stained by a standard hematoxylin/eosin (HE) staining for cytological evaluation of polymorphonuclear cells infiltration.

### Statistical Evaluations

The cytotoxicity data were expressed as the percentage of viability, where 100% represented the treatments with vehicle (0.1% DMF or 0.1% DMSO). The cytotoxicity data from the cancer cell lines were acquired from three independent experiments (conducted in triplicate) using cells from different passages. The IC_50_ values were calculated from viability curves. The results are presented as arithmetic means±standard error of the mean (S.E.). The significance of the differences between the results was assessed by the ANOVA analysis with p<0.05 considered to be significant (QC Expert 3.2, Statistical software, TriloByte Ltd., Pardubice, Czech Republic).

The statistically significant differences between individual groups during anti- inflammatory testing were evaluated using a one-way ANOVA test for statistical analysis, followed by Tukey's *post-hoc* test for multiple comparisons. GraphPad Prism 5.02 (GraphPad Software Inc., San Diego, CA, USA) was used to perform the analysis. The results are presented as arithmetic means±S.E. values.

### Interactions of the Complexes with Sulfur-containing Biomolecules

The interactions of selected gold(I) complexes **2** and **4**, involving the isopropyloxy and benzyloxy substituent on C6 of 9-deazapurine, with the sulfur-containing biomolecules (i.e. L-cysteine and reduced glutathione) were studied by ESI+ mass spectrometry (ESI+ MS). The experiments were carried out using a Thermo Scientific LTQ Fleet Ion-Trap mass spectrometer, in positive ionization mode. The reactions of the representative complexes **2** and **4** were performed in methanol/water mixture (1∶1, v/v) containing the physiological concentrations of L-cysteine and glutathione (at the final concentrations of 290 µM, and 6 µM [63], respectively). The reference system consisted of the solutions of complexes (20 µM) in methanol/water mixture (1∶1, v/v). The flow injection analysis (FIA) method was utilized to introduce the reaction system (5 µL spikes) into the mass spectrometer and pure acetonitrile was used as a mobile phase. The ESI-source was set-up as follows: source voltage (5 kV), the vaporizer temperature (160°C), the capillary temperature (250°C), the sheath gas flow (30 L/min), and auxiliary gas flow rate (10 L/min). The system was calibrated as stated in the manufactured specifications and no further tuning was needed.

## Results and Discussion

### General Properties of Gold(I) Complexes 1–5

**Figure 2:**
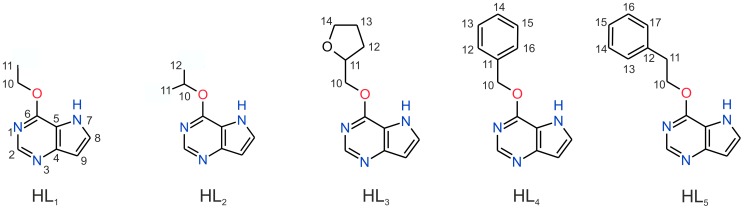
Schematic representations of *O*-substituted 9-deazahypoxanthine derivatives (HL_1–5_) used as the ligands in complexes 1–5.

**Figure 3:**
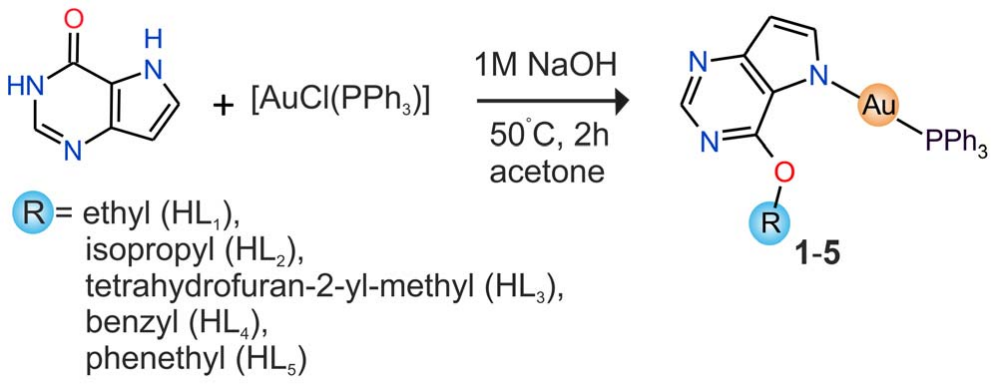
A pathway for the preparation of complexes 1–5.

The pale yellow gold(I) complexes of the composition [Au(L*_n_*)(PPh_3_)] (**1**–**5**), where L*_n_* stands for a deprotonated form of the appropriate *O*-substituted 9-deazahypoxanthine derivative (HL_1–5_, Figure 2), were prepared in relatively high yields of 60–75%, as shown in Figure 3. The complexes **1**–**5** are very soluble in *N,N'*-dimethylformamide, dimethyl sulfoxide and acetone, soluble in alcohols and very slightly soluble in water. Their composition and structure were proved using a variety of physical techniques, mainly by single crystal X-ray analysis in the case of [Au(L_2_)(PPh_3_)] (**2**). Moreover, their thermal stability was determined by TG/DTA techniques, using complexes **2** and **4** as representative samples (see Figure S1 in Information S1). ESI+ MS spectra of **1**–**5** in methanol solutions (10^−5^ M) showed the [Au(L*_n_*)(PPh_3_) + H]^+^ molecular peaks of all the studied complexes at *m/z* 622.2 (**1**), 636.2 (**2**), 678.1 (**3**), 684.2 (**4**) and 698.1 (**5**). The sodium adducts of [Au(L*_n_*)(PPh_3_) + Na]^+^ with usually lower intensity than the molecular peaks were also observed in the mass spectra (for more detailed information about ESI+ mass spectra see Figure S2 in Information S1). The mid-IR spectra confirmed the presence of both types of the ligands in the complexes, as may be demonstrated by peaks observed at 3077–3018, 1593–1589 and 1545–1470 cm^−1^, which could correspond to the ν(C–H)_ar_, ν(C–N)_ring_, and ν(C–C)_ring_ stretching vibrations, respectively. In the far-IR spectra, the bands detected at *ca*. 509–502 cm^−1^ and 310–289 cm^−1^ can be assigned to the ν(Au–N), and ν(Au–P) stretching vibrations, respectively, [64]–[65] (for more detailed information about FT-IR spectra see Information S1).

The ^1^H and ^13^C spectra were obtained for all the complexes **1–5** and free ligands HL*_n_*. The interpretation of the spectra clearly confirmed the presence of the organic molecules, i.e. deprotonated *O*-substituted 9-deazahypoxanthine derivatives (L*_n_*) and triphenylphosphine (PPh_3_), in the presented complexes (see Figure S3 and S4 in Information S1) and the comparison of chemical shifts (δ) in the NMR spectra of free compounds HL*_n_* and complexes **1–5**, which are further discussed as coordination shifts, Δδ  =  δ_complex_ − δ_ligand_; ppm, provided information about the possible coordination mode of these ligands to the metal centre (Table 1). In general, parts of NMR spectra regarding the HL*_n_* ligands in the complexes were qualitatively similar to the spectra of the corresponding free HL*_n_*, except for the signals of the atoms lying in the vicinity of the coordination site, i.e. the N7 atom, whose chemical shifts changed significantly. Accordingly, the greatest changes were detected for the C5 and C8 atoms, adjacent to the N7 coordination site and shifted by 7.44–7.65 ppm, and 10.09–10.19 ppm downfield, respectively. In the proton NMR spectra, the most shifted signals were found for the C2H and C8H atoms (0.14–0.16 ppm, and 0.08–0.10 ppm upfield, respectively). It is noteworthy to mention that the ^1^H NMR spectra of **1**–**5** also showed the absence of the signal corresponding to the N7H proton with respect to NMR spectra of free HL*_n_*. Further, the signals of triphenylphosphine ligand in **1**–**5** were detected in the region around 7.70 ppm and 130 ppm in the proton, and carbon spectra, respectively, with relative integral intensity corresponding to 15 protons of this compound. All the above mentioned chemical shifts of the signals observed in the NMR spectra of the herein reported complexes indirectly confirmed the presence of one molecule of PPh_3_ and deprotonated L*_n_* molecule in **1**–**5**, and the coordination of L*_n_* through the N7 atom to the metal centre as it was determined using X-ray analysis of **2**.

**Table 1:** ^1^H and ^13^C NMR coordination shifts (Δδ  =  δ_complex_ – δ_ligand_; ppm) of *O*-substituted 9-deazahypoxanthine moiety atoms in complexes **1**–**5**.

	^1^H NMR			^13^C NMR					
	C2H	C8H	C9H	C2	C8	C9	C4	C5	C6
**1**	−0.14	−0.10	−0.04	−2.17	10.10	−0,79	1.07	7,44	0,82
**2**	−0.16	−0.09	−0.03	−2.15	10.09	−0.76	1.07	7.64	0.86
**3**	−0.16	−0.10	−0.05	−2.16	10.16	−0.78	1.04	7.58	0.77
**4**	−0.16	−0.10	−0.02	−2.21	10.19	−0.72	1.14	7.65	0.74
**5**	−0.14	−0.08	−0.01	−2.20	10.13	−0.67	1.36	7.65	0.71

### Crystal Structures of 6-phenethyloxy-9-deazapurine (HL_5_) and [Au(L_2_)(PPh_3_)] (2)

**Figure 4:**
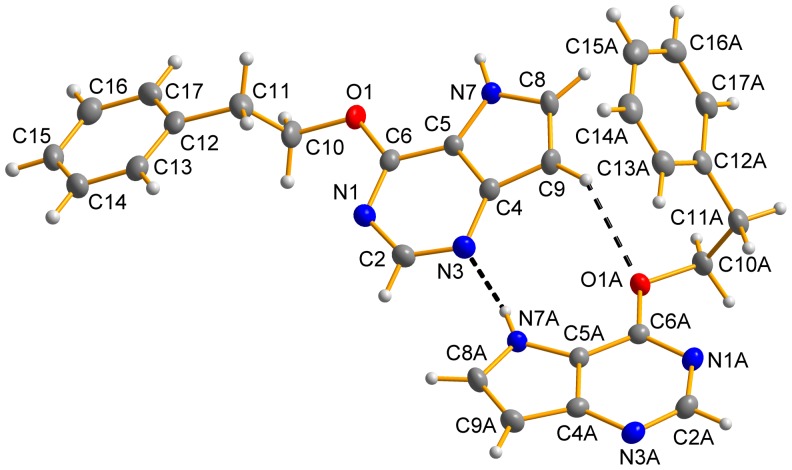
The molecular structure of 6-phenethyloxy-9-deazapurine (HL_5_), showing the atom numbering scheme and N–H⋅⋅⋅N and C–H⋅⋅⋅O non-covalent bonding (dashed lines). Non-hydrogen atoms are displayed as ellipsoids at the 50% probability level.

**Figure 5:**
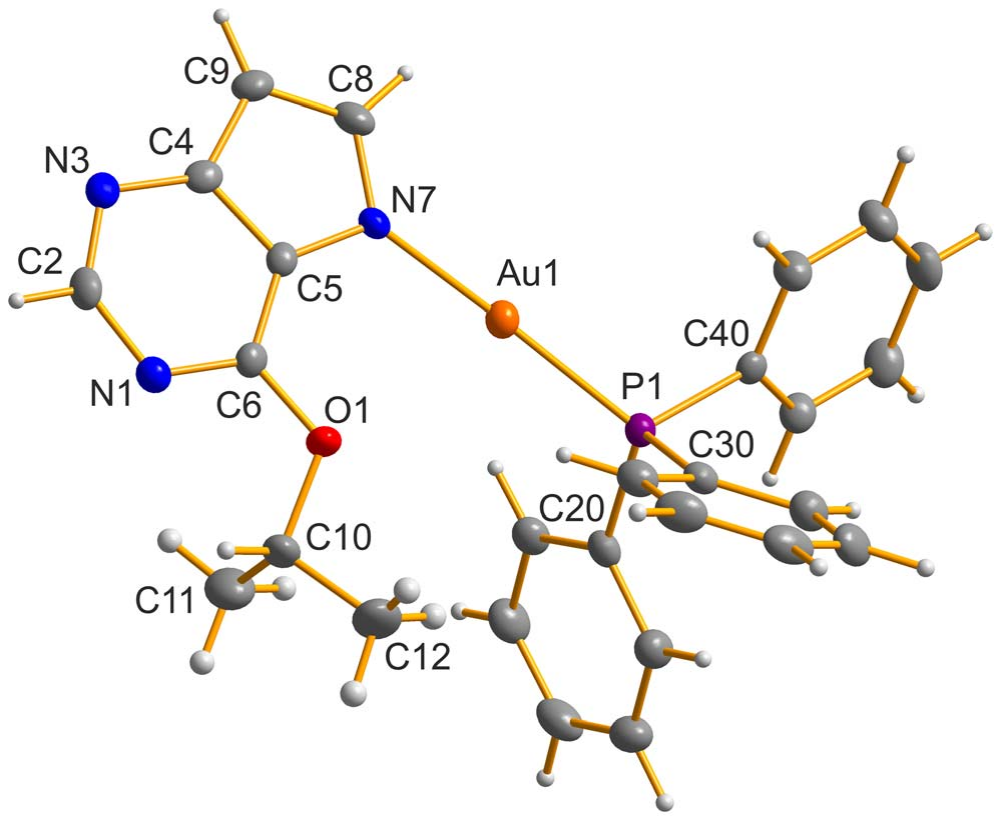
The molecular structure of [Au(L_2_)(PPh_3_)] (2), showing the atom numbering scheme. Non-hydrogen atoms are displayed as ellipsoids at the 50% probability level.

The crystals of 6-phenethyloxy-9-deazapurine (HL_5_) and [Au(L_2_)(PPh_3_)] (**2**), where HL_2_ = 6-isopropyloxy-9-deazapurine, suitable for the single crystal X-ray analysis were obtained by slow evaporation of the saturated acetonitrile, and acetone solution, respectively. The molecular structures of HL_5_ and **2** are depicted in Figures 4, and 5, respectively. The crystal data and structure refinements (see Table S1 in Information S1), selected bond lengths and angles (see Tables S2 and S3 in Information S1) and parameters of selected non-covalent contacts (see Tables S4 and S5 in Information S1) are listed in Information S1. The molecular structure of HL_5_ consists of two crystallographically independent molecules within the asymmetric unit (discussed as HL_5_ and HL_5A_), which are mutually connected through the N7–H···N3 and C9–H···O1 non-covalent contacts connecting also both individual molecules into one dimensional supramolecular chains (see Figure S5 in Information S1), which are mutually connected through C–H···C, C–H···N and C···C interactions. Parameters of selected non-covalent contacts are given in Table S4 in Information S1.

Single crystal X-ray analysis of **2** confirmed coordination mode of 6-isopropyloxy-9-deazapurine (HL_2_) to the gold(I) centre in the complexes **1**–**5**, as suggested by ^1^H and ^13^C NMR spectrometry. As shown in Figure 5, the gold(I) atom of **2** is two-coordinated in a slightly distorted linear fashion [N7–Au1–P1 = 176.35(6)°], with the {NP} donor set formed by the N7 atom of 6-isopropyloxy-9-deazapurine and P1 atom of triphenylphosphine. The Au1–N7 and Au1–P1 bond lengths of **2**, i.e. 2.041(2) Å, and 2.2272(7) Å, respectively, are comparable with those found in the compounds involving the same N–Au–P structural motif deposited in Cambridge Structural Database (CSD, ver. 5.35, February 2014 update), which were found to lie in the range of 1.91–2.32, and 2.17–2.29 Å, respectively [66]. Based on the search within CSD, the mean N–Au–P angle is around 175° in about 260 gold(I) complexes. Further, the crystal structure of **2** is stabilized by C–H···C and C–H···N non-covalent interactions (see Table S5 in Information S1).

### *In Vitro* Cytotoxicity

In order to analyse the potential of the gold(I) compounds as anticancer agents, complexes **1**–**5** were studied by the MTT assay for their *in vitro* cytotoxic activity against a variety of human cancer cell lines, i.e. MCF7 breast carcinoma, HOS osteosarcoma, A549 lung carcinoma, G361 malignant melanoma, HeLa cervix epitheloid carcinoma, A2780 ovarian carcinoma, A2780R ovarian carcinoma resistant to *cisplatin* and 22Rv1 prostate carcinoma. For comparison purposes, the cytotoxic activity of the commercially used drug *cisplatin* and other relevant compounds, i.e. AuCl, HAuCl_4_ and free HL*_n_*, was evaluated by using the same experimental conditions. As for the obtained results regarding the relevant compounds they were found as inactive up to the concentration of 50 µM, except for HAuCl_4_ which showed a moderate effect only on G361 (IC_50_ = 38.1±2.3 µM). *In vitro* anticancer activity data are summarized in Table 2.

**Table 2:** The results of *in vitro* cytotoxicity of complexes1–5 and *cisplatin* against variety of human cancer and healthy cell lines.

Compound	Human Cell Line									
	MCF7	HOS	A549	G361	HeLa	A2780	A2780R	22Rv1	THP-1	HEP220
**1**	3.1±0.2*	3.3±0.1*	20.7±0.1	3.5±0.2*	22.0±0.6	4.8±0.1*	5.2±0.3*	3.5±0.1*	0.8±0.1	24.0±1.9
**2**	0.6±0.1*	0.9±0.2*	17.2±0.7	3.4±0.1*	16.0±0.2	4.0±0.2*	5.3±0.4*	4.0±0.1*	0.8±0.1	18.5±1.5
**3**	2.2±0.2*	4.0±0.6*	21.4±0.2	3.4±0.2*	20.7±0.2	4.6±0.3*	5.3±0.4*	21.0±0.7	1.0±0.1	24.1±2.4
**4**	4.0±0.9*	1.8±0.3*	>20	3.5±0.2*	14.3±0.2	4.6±0.2*	5.1±0.3*	3.5±0.2*	1.7±0.1	23.7±2.1
**5**	>50	2.9±0.1*	18.3±0.5	3.5±0.2*	22.8±0.5	4.4±0.3*	5.0±0.3*	3.7±0.1*	1.4±0.1	19.0±1.8
*cisplatin*	17.9±1.2	20.5±0.1	>50	5.3±0.2	>50	11.5±0.5	27.0±1.5	26.9±1.2	n.d.	>50

Cells were treated with the tested compounds for 24 h; measurements were performed in triplicate, and cytotoxicity experiments were repeated on three different cell passages; data are expressed as IC_50_±S.E. (µM).

n.d. – not determined; asterisk (*) symbolizes significant difference (p<0.05) in *in vitro* cytotoxicity of **1**–**5** as compared with *cisplatin*.

As can be seen from Table 2, the complexes **1**–**5** were found to be anticancer effective against all the cancer cell lines tested, with IC_50_≈0.6–22.8 µM. However, the complexes revealed selectively and significantly higher anticancer activity on MCF7, HOS, G361, A2780, A2780R and 22Rv1 as compared to *cisplatin*, with IC_50_≈0.6–5.3 µM, except for complex **5** on MCF7 (IC_50_ >50 µM) and complex **3** on 22Rv1 (IC_50_ = 21.0±0.7 µM). Moreover, the *in vitro* cytotoxicity testing of **1**–**5** evaluated against the A2780 and A2780R cell lines showed a similar pattern of response across the parental and resistant sub-lines and allowed the calculation of resistance factor (RF) values (defined as the ratio between the IC_50_ values calculated for the resistant cells and those arising from the sensitive ones; IC_50_(A2780R)/IC_50_(A2780)) ranging from 1.1 to 1.3 in comparison with the value obtained for *cisplatin*, which equals to 2.4.

All the gold(I) complexes **1**–**5** were also evaluated for *in vitro* cytotoxicity against the THP-1 cells (Table 2). The complexes showed a strong *in vitro* cytotoxic action with the IC_50_ values in the range of 0.8–1.7 µM, comparable to Auranofin (IC_50_ = 0.9±0.1 µM). An interesting finding is the fact that all the complexes showed the hormetic effect at very low concentrations of ca. 0.3 µM. This is in accordance with the behaviour of the previously reported gold(I) complexes containing derivatives 6-benzylaminopurines (HBap) of the composition [Au(Bap)(PPh_3_)] [38], [67].

With the aim to reveal the influence of complexes **1**–**5** on healthy tissues, the *in vitro* cytotoxicity against primary culture of human hepatocytes was evaluated. It has been found that the complexes **1**–**5** reached up to 30-times lower cytotoxicity (complex **2** on MCF7 *vs.* HEP220) against human healthy cells in comparison to cancer cell lines. A relatively broad concentration range between anti-proliferative and cytotoxicity on healthy cells shows on real applicability of the complexes, although we are aware of the fact that next deeper biological studies are needed. The respective data are included in Table 2.

### *In Vitro* Anti-inflammatory Activity

**Figure 6:**
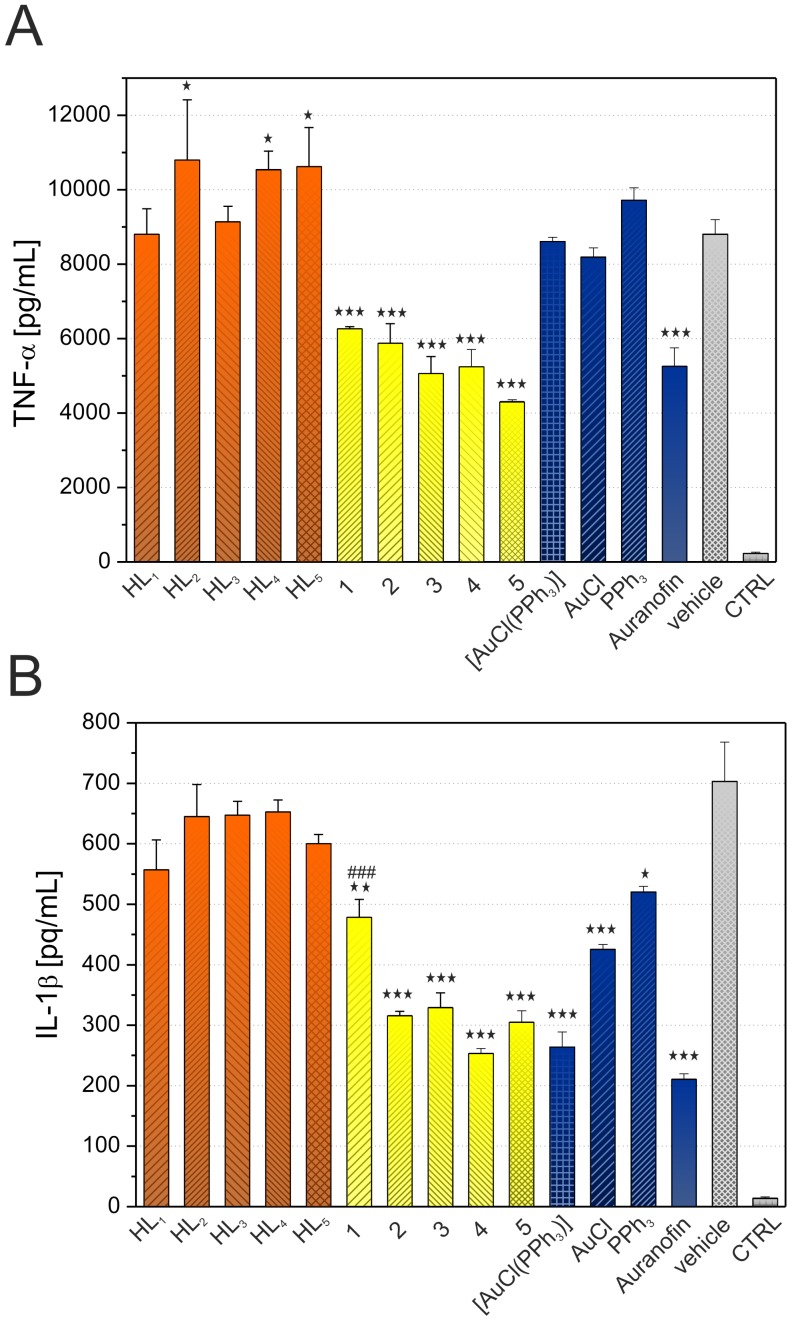
Effects of complexes 1–5, Auranofin, and other relevant compounds on LPS-induced TNF-α (A) and IL-1β (B) secretion. The cells were pretreated with the tested compounds (300 nM) or the vehicle (0.1% DMSO) only. After 1 h of the incubation, the inflammatory response was induced by LPS [except for the control cells (CTRL)]. The secretion was measured 24 h after the LPS addition. The results are expressed as means±S.E. of three independent experiments. Significant difference in comparison to: * vehicle-treated cells (p<0.05), ** vehicle-treated cells (p<0.01), *** vehicle-treated cells (p<0.001), ### Auranofin-treated cells (p<0.001) (determined only for complexes **1**–**5**).

For the evaluation of *in vitro* anti-inflammatory activity, the ability of the complexes **1**–**5** to decrease the production of pro-inflammatory cytokines TNF-α and IL-1β in LPS-stimulated macrophage-like cells was determined. The results showed that all the tested complexes significantly decreased the production of both pro-inflammatory cytokines (Figure 6), however the discrepancy was found between the abilities of complexes to influence the secretion of IL-1β in comparison with TNF-α. This observation is in accordance with our previous results regarding the anti-inflammatory activities of gold(I) complexes of the type [Au(L)(PPh_3_)], where L represents other types of *N*-donor ligands, e.g. different adenine derivatives [38], [67]. Concretely, the effect of complexes **1**–**5** on the secretion of pro-inflammatory cytokine TNF-α was comparable with Auranofin (Figure 6A), while none of the reference compounds, such as AuCl, HL*_n_*, [AuCl(PPh_3_)] and PPh_3_, exhibited any expected effect. Moreover, the compounds HL_2_, HL_4_, and HL_5_ were found to stimulate the production of this cytokine. This indicates that only the whole complexes are able to diminish the production of TNF-α. More complicated situation was found in the case of evaluation of secretion of pro-inflammatory cytokine IL-1β influenced by complexes **1**–**5**. Although complexes **1**–**5** significantly attenuated the secretion of this cytokine, only **2**–**5** had a similar effect as Auranofin with the secretion level decreased to 50–70% (Figure 6B). Further, compound **1** diminished the level of IL-1β only by 32%, similarly as free triphenylphosphine. To understand the role of individual constituent as a part of complexes in vanquished production of IL-1β, the other reference compounds, as free molecules of HL*_n_*, PPh_3_, AuCl and [AuCl(PPh_3_)], were also evaluated. It can be pointed out that the compounds PPh_3_ and AuCl decreased the production of IL-1β by a manner comparable to free ligands.

**Figure 7:**
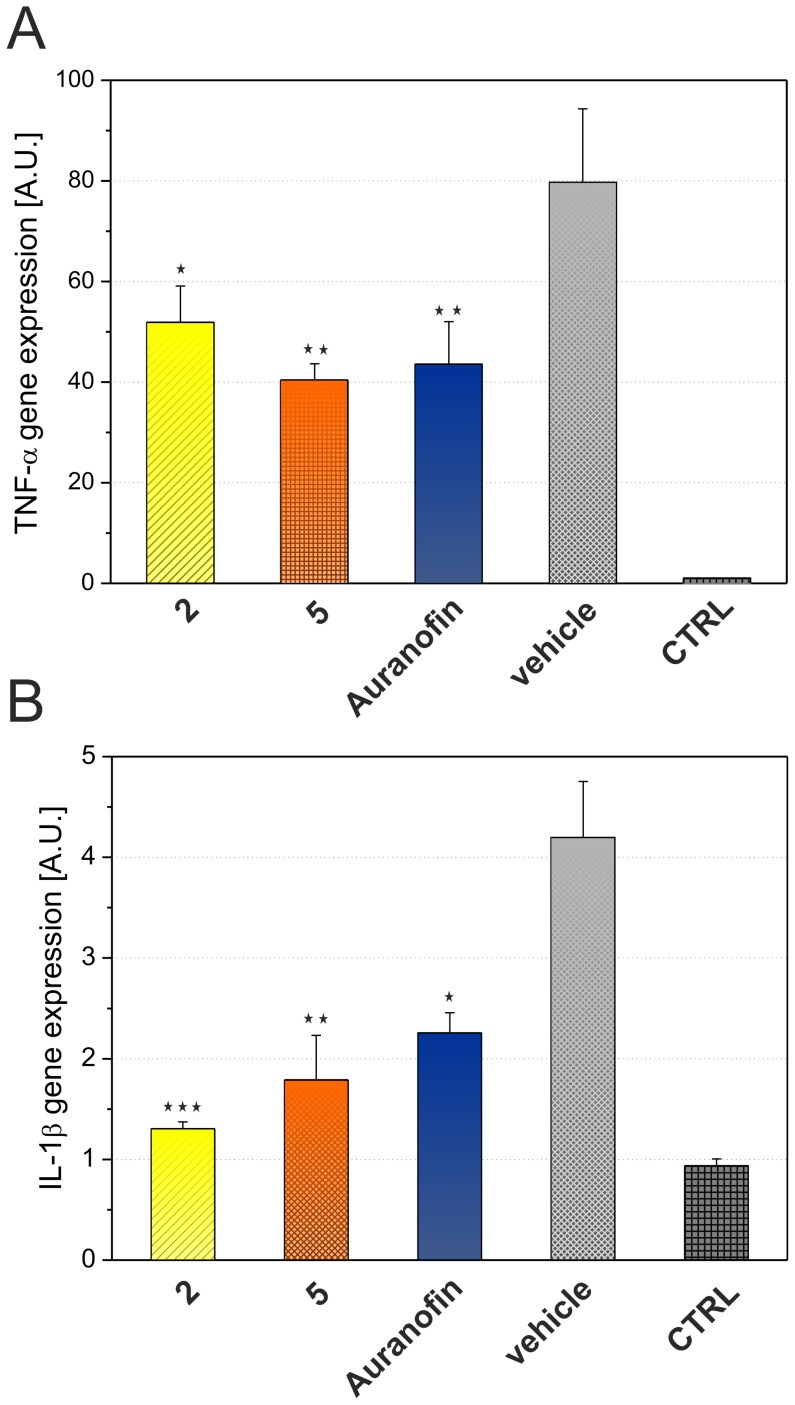
Effects of complexes 2 and 5, and Auranofin on gene expression of TNF-α (A) and IL-1β (B). THP-1 macrophages were pretreated with compounds **2**, **5**, and Auranofin at the concentration of 300 nM or the vehicle (0.1% DMSO) only. After 1 h of the incubation, the inflammatory response was induced by LPS [except for the control cells (CTRL)]. After 2 h, the level of TNF-α and IL-1β mRNA was evaluated by RT-qPCR. The amount of cytokines mRNA was normalised to β-actin mRNA. The results are expressed as means ± S.E. of three independent experiments. A.U.  =  arbitrary unit. * significant difference in comparison to vehicle-treated cells (p<0.05), ** significant difference in comparison to vehicle-treated cells (p<0.01), *** significant difference in comparison to vehicle-treated cells (p<0.001).

**Figure 8:**
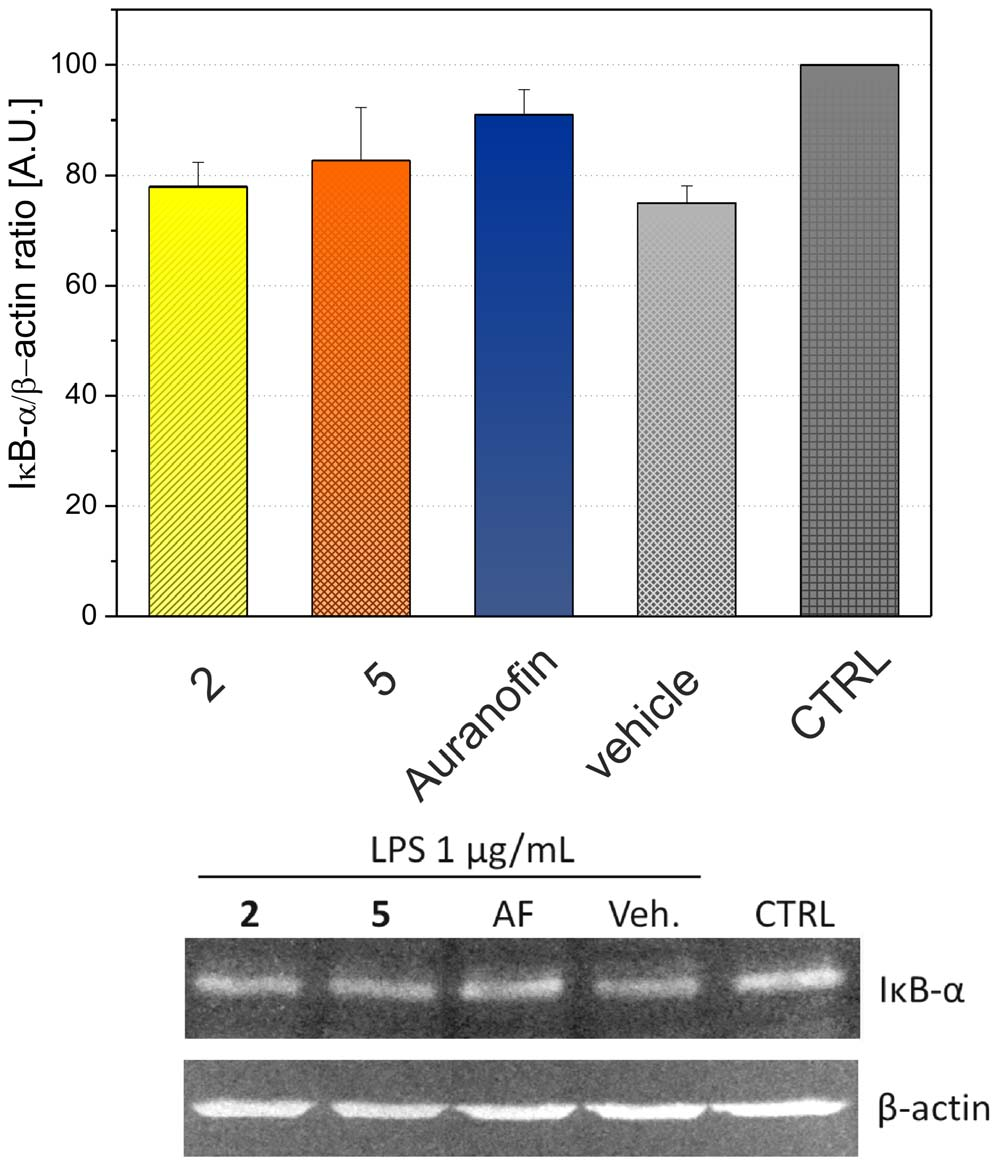
Effects of complexes 2 and 5, and Auranofin on the LPS-induced degradation of IκB-α. The cells were pretreated with tested compounds (300 nM) or the vehicle (Veh., DMSO) only. After 1 h of incubation, the inflammatory response was induced by LPS [except for the control cells (CTRL)]. The levels of IκB-α and β-actin were measured 30 min after LPS treatment. The graph indicates the IκB-α/β-actin ratio. The results are expressed as means ± S.E. of three independent experiments. The blots show the representative results from three independent experiments.

To evaluate whether the secretion of TNF-α and IL-1β is attenuated by post-translation or by pre-translation mechanism, the influence of selected complexes **2** and **5** (these were chosen in connection with their structural diversity in *O*-substitution at the C6 atom of the ligand) on gene expression was assessed on the level of mRNA [68]. Both the complexes as well as Auranofin were able to significantly reduce the transcription of these cytokines (Figure 7). Pro-inflammatory cytokines TNF-α and IL-1β are under the transcription control of the transcription factor NF-κB, therefore the effect of the complexes on this signalling pathway was examined. Particularly, the effect on IκB degradation was evaluated. As shown in Figure 8, the complexes **2** and **5** were able to block moderately the IκB degradation as effectively as Auranofin. These results indicate that the tested complexes attenuate the pro-inflammatory cytokine production, at least in part, due to the blocking of the NF-κB signalling pathway through the inhibition of IκB degradation. This observation is in the concordance with previous findings, in which Auranofin and other gold-containing complexes are able to bind to cysteine residues of IκB kinase (IKK) and thus block its function [69].

### *In Vivo* Anti-inflammatory Activity and *Ex Vivo* Histological Evaluation

**Figure 9:**
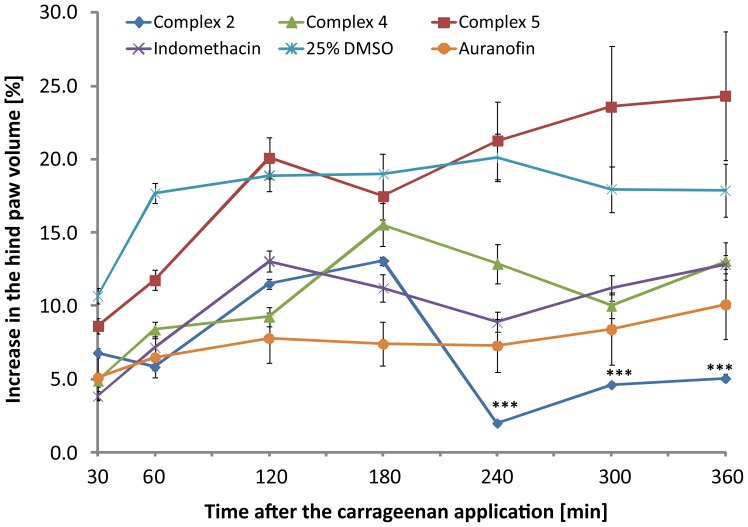
The time-dependent profile of antiedematous activity of complexes 2, 4 and 5, and Indomethacin.

Based on the promising results of *in vitro* experiments, the complexes **2**, **4** and **5** were subjected to *in vivo* tests of anti-inflammatory activity using the carrageenan-induced hind paw edema model. This model evaluates the effect of the tested complexes on the acute inflammatory process induced by the polysaccharide carrageenan injection. The main symptom of this process is the formation of edema, which is assessed plethysmometrically. The clinically used non-steroidal anti-inflammatory drug Indomethacin was used as a primary standard for anti-inflammatory activity, and pharmacological profiles of the tested complexes were compared to the previously published results of gold-containing drug Auranofin [38]. In the experiments, we used the dosages of the tested compounds equivalent by the content of gold to 10 mg/kg dosage of Auranofin. The complexes were applied intraperitonealy in the form of the fine suspension in 25% DMSO (v/v in water) 30 min before the intraplantar injection of carrageenan. A reference standard of Indomethacin was applied in the dose of 5 mg/kg [70]. The comprehensive overview of antiedematous activity profiles of the tested compounds is summarized in Figure 9.

The results of antiedematous activity showed very similar pharmacological profiles of complexes **2** and **4** with the reference drug Indomethacin up to the 180 min after the application of carrageenan. After this time point, the complex **2** showed significant increase in biological activity, leading to the amelioration of the inflammatory response, which resulted in elimination of hind paw swelling. In this time period, the antiedematous effect of complex **2** was found to be even better than that of gold-containing metallodrug Auranofin and showed a significant difference at the probability level p<0.001. With respect to the structural similarity of all tested complexes and expected similar mechanism of action, their efficacy is probably dependent on the bioavailability, while the molecular weight might be a key parameter in this matter. This hypothesis is supported by the results of antiedematous activity of complex **5** (having the highest molecular weight) that was identified as inactive.

**Figure 10:**
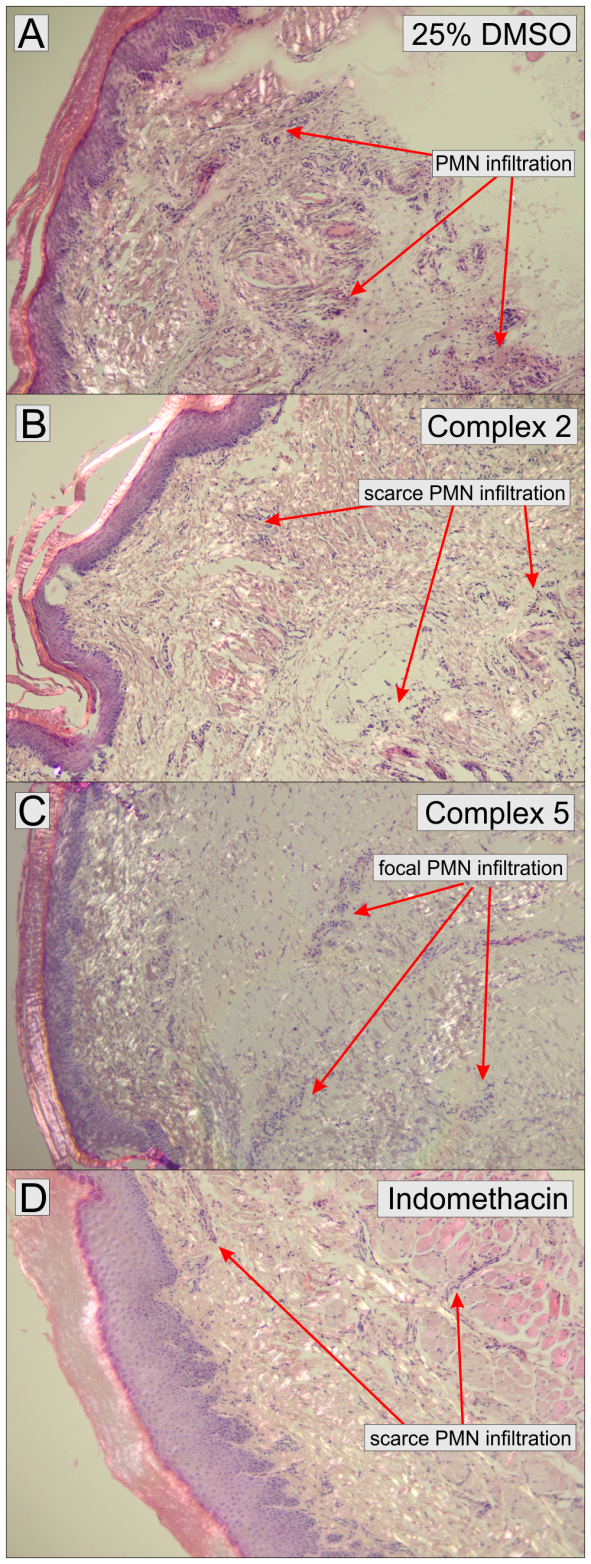
Histological evaluation of inflammatory response in tissue sections of the hind paw, stained with Hematoxylin – eosin (40x magnification). Tissue exposed to 25% DMSO (control; **A**) and complex **5** (**C**) with the acute inflammatory reaction dermis and hypodermis with a massive infiltrate of neurophils (PMN); tissue exposed to complex **2** (**B**) and Indomethacin (**D**) with the inflammatory reaction in the hypodermis with scarce PMN infiltrate.

To assess the tissue consequences connected with the reduction of inflammation caused by the tested compounds after the intraplantar injection of carrageenan, the histopathological observations were made on the tissue sections obtained from the laboratory animals after the plethysmometric experiments were finished. All animals were sacrificed by cervical dislocation, and immediately after that, the tissue samples were taken from the plantar area of hind paws. The histopathological changes in tissues, stained by the standard HE staining (see Figure 10), were evaluated by the presence of the inflammation infiltrate, which contained mainly neutrophils (polymorphonuclear cells - PMN). These changes provided evidence of the acute inflammation, which were manifested by the massive presence of PMN cells, in the samples from the control group (see Figure 10A) and the group pretreated with complex **5** (see Figure 10C), which showed the lowest antiedematous effect in plethysmometrical evaluation. On the other hand, the PMN distribution was mainly scarce and diffuse in samples obtained from the Indomethacin (see Figure 10D) and complex **2** (see Figure 10B) treated groups. Both these substances significantly decreased the inflammatory reaction.

### Interactions with Cysteine and Reduced Glutathione Analysed by ESI MS

The gold(I) species prefer to form the strong coordination bonds with soft Lewis base ligands, i.e. thiolate or selenolate ions, or phosphine derivatives. It is a well known fact, that Au(I) complexes bind to selanyl- and sulfanyl- groups of biomolecules, such as amino acid cysteine (Cys), small proteins, such as glutathione (GSH), and high molecular weight proteins (e.g. selenium flavoproteins, serum albumin or globulins [71]) by the ligand exchange mechanism. The exchange of *N*-ligands for *S*-ligands occurs relatively fast (within 20 minutes when interacting with albumin and globulins in the blood [72]), while the *P*-ligand exchange proceeds much more slowly, involving a much more complicated mechanism. It seems that in this mechanism the cooperative effects of adjacent thiolato or selenolato ligands in the neighbourhood of the interaction site play an important role. As such, the described ligand exchange is interpreted as one of the molecular mechanisms of incorporation of gold into the active site of selenium-containing flavoreductases, such as thioredoxin reductase [73]. In the scope of this work, we strived to uncover the molecular behaviour of anti-inflammatory active complex **2** (applied in the concentration of 20 µM, corresponding approximately to the highest therapeutic blood levels of gold during chrysotherapy [74]) in biologically relevant conditions using a mixture of cysteine (at 290 µM concentration) and reduced glutathione (at the 6 µM concentration) [63].

**Figure 11:**
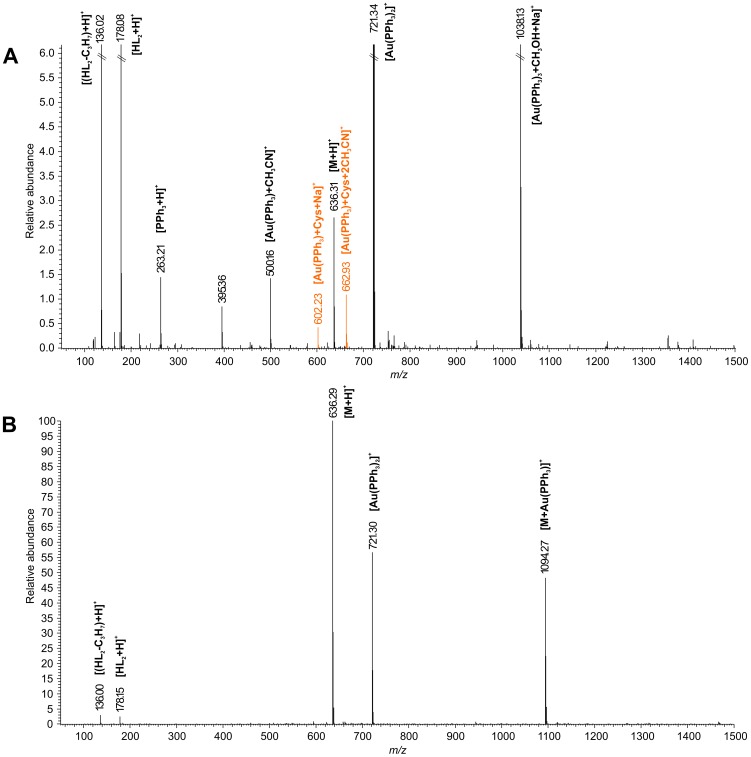
ESI+ MS spectra of the interacting system containing cysteine + glutathione + complex 2 in the water/methanol (1∶1 v/v) mixture (A), and the solution of complex 2 in the water/methanol (1∶1 v/v) mixture (B).

Based on the results of the ESI-MS experiments, we confirmed that complex **2** is able to react with the used sulfhydryl-containing substances quite rapidly (the interaction intermediates were detected within 1 h) by the ligand-exchange mechanism associated with the substitution of the *N*-ligand (L_n_) by the cysteine or glutathione molecule. This mechanism was confirmed by the emergence of the signals at 602.23 *m/z*, and 662.93 *m/z*, corresponding to the [Au(PPh_3_)+Cys+Na]^+^, and [Au(PPh_3_)+Cys+2CH_3_CN]^+^ intermediates, respectively (see Figure 11).

In concordance with the above mentioned suggestion and in accordance with the previously reported behaviour of some Au(I) complexes in water-containing solutions [26], the mass spectra of the reacting systems involving sulfur-containing molecules and also the reference solutions of complexes revealed a considerable instability of the complexes demonstrated by the appearance of the intensive ion at 721.34 *m/z*, corresponding to the [Au(PPh_3_)_2_]^+^ intermediate, and other ionic species involving the residue Au-PPh_3_ (*i.e.* [Au(PPh_3_)_3_+CH_3_OH+Na]^+^ at 1038.30 *m/z*, and [Au(PPh_3_)+CH_3_CN]^+^ at 500.16 *m/z*), the free HL_n_ molecules ([HL_2_+H]^+^ at 178.08 *m/z*, or the free triphenylphosphine residue (*i.e.* [PPh_3_+H]^+^ at 263.24 *m/z*.

## Conclusions

A series of gold(I) complexes of the general formula [Au(L*_n_*)(PPh_3_)] (**1**–**5**) (involving *O*-substituted 9-deazahypoxanthine derivatives; HL*_n_*) is reported. The complexes were thoroughly structurally characterized and their anticancer (*in vitro*) and anti-inflammatory (*in vitro* and *in vivo*) activities were evaluated. The cytotoxicity results revealed that the complexes are significantly anticancer effective against MCF7, HOS, A2780, A2780R and 22Rv1, with IC_50_≈0.6–5.3 µM, whereas the complex **2** was identified as the most active, being at least 20-times more efficient as *cisplatin* on the MCF7 and HOS cell lines. On the other hand, the complexes showed up to 30-times lower cytotoxicity against healthy cells (human hepatocytes, HEP220) as compared with cancer cells. The results of *in vitro* and *in vivo* anti-inflammatory activity screening indicated that complexes **2** and **4** show significant anti-inflammatory effects on both levels, comparable with the commercially used drug Auranofin. It may be concluded, in connection with the overall positive findings regarding the biological testing, that the [Au(L*_n_*)(PPh_3_)] **1**–**5** complexes could represent usable alternatives to anticancer (*cisplatin*) as well as anti-inflammatory (Auranofin) metallodrugs of major diseases negatively affecting humankind.

## Supporting Information

Information S1 Synthesis, elemental analysis and ESI MS, FT-IR, ^1^H and ^13^C NMR data for HL_1–5_ as well as the results of elemental analysis, TG/DTA, ESI MS, FT-IR, ^1^H and ^13^C NMR experiments assigned to complexes **1**–**5** are given in Information S1. **Figure S1**. TG/DTA curves of the complexes **2** and **4**. **Figure S2**. ESI+ MS spectrum of **4**. **Figure S3**. ^1^H and ^13^C NMR spectra of **2**. **Figure S4**. ^1^H–^13^C HMQC NMR spectra of **2**. **Figure S5**. A part of the crystal structure of HL_5_. **Table S1**. Crystal data and structure refinements for HL_5_ and **2**. **Table S2**. Selected bond lengths and angles in HL_5_. **Table S3**. Selected bond lengths and angles in complex **2**. **Table S4**. Selected non-covalent contacts in the crystal structure of HL_5_. **Table S5**. Selected non-covalent contacts in the crystal structure complex **2**.
(DOCX)

## References

[1] Gielen M, Tiekink ERT (2005) Metallotherapeutic Drugs and Metal-based Diagnostic Agents: The Use of Metals in Medicine. London: John Wiley and Sons, Ltd., Chichester, England.

[2] Farrer NJ, Sadler PJ (2011) Bioinorganic Medicinal Chemistry. In: Alessio E, editors. Weinheim: Wiley-VCH, Germany, pp. 1–48.

[3] Sigler JW, Bluhm GB, Duncan H, Sharp JT, Ensign DC, McCrum WR (1974) Gold Salts in the Treatment of Rheumatoid Arthritis: A Double-Blind Study. Ann Intern Med 80: 21–26. 4204032 10.7326/0003-4819-80-1-21

[4] Williams HJ, Ward JR, Reading JC, Brooks RH, Clegg DO, et al (1992) Comparison of Auranofin, methotreaxate, and the combination of both in the treatment of rheumatoid arthritis. A controlled clinical trial. Arthritis Rheum 35: 259–269. 1536666 10.1002/art.1780350304

[5] Kean WF, Kean IRL (2008) Review: Clinical pharmacology of gold. Inflammopharmacology 16: 112–125. 18523733 10.1007/s10787-007-0021-x

[6] Kean WF, Hart L, Buchanan WW (1997) Auranofin. Br J Rheumatol 36: 560–572. 9189058 10.1093/rheumatology/36.5.560

[7] Eisler R (2003) Chrysotherapy: a synoptic review. Inflamm Res 52: 487–501. 14991077 10.1007/s00011-003-1208-2

[8] Bruijnincx PCA, Sadler PJ (2008) New trends for metal complexes with anticancer activity. Curr Opin Chem Biol 12: 197–206. 18155674 10.1016/j.cbpa.2007.11.013 PMC2923029

[9] Berners-Price SJ, Filipovska A (2008) The Design of Gold-Based, Mitochondria-Targeted Chemotherapeutics. Aust J Chem 61: 661–668.

[10] Hambley TW (2007) Metal-Based Therapeutics. Science 318: 1392–1393. 18048674 10.1126/science.1150504

[11] Ott I (2009) On the medicinal chemistry of gold complexes as anticancer drugs. Coord Chem Rev 253: 1670–1681.

[12] Milacic V, Dou QP (2009) The tumor proteasome as a novel target for gold(III) complexes: implications for breast cancer therapy. Coord Chem Rev 253: 1649–1660. 20047011 10.1016/j.ccr.2009.01.032 PMC2675785

[13] Nardon C, Boscutti G, Fregona D (2014) Beyond platinums: gold complexes as anticancer agents. Anticancer Res 34: 487–492. 24403506

[14] Casini A, Messori L (2011) Molecular mechanisms and proposed targets for selected anticancer gold compounds. Curr Top Med Chem 11: 2647–2660. 22039866 10.2174/156802611798040732

[15] Che CM, Sun RW (2011) Therapeutic applications of gold complexes: lipophilic gold(III) cations and gold(I) complexes for anti-cancer treatment. Chem Commun (Camb). 47: 9554–9560. 10.1039/c1cc10860c 21674082

[16] Madeira JM, Gibson DL, Kean WF, Klegeris A (2012) The biological activity of auranofin: implications for novel treatment of diseases. Inflammopharmacology 20: 297–306. 22965242 10.1007/s10787-012-0149-1

[17] Mirabelli CK, Johnson RK, Sung CM, Faucette L, Muirhead K, et al (1985) Evaluation of the *in vivo* antitumor activity and *in vitro* cytotoxic properties of auranofin, a coordinated gold compound, in murine tumor models. Cancer Res 45: 32–39. 3917372

[18] Simon TM, Kunishima DH, Vibert GJ, Lorber A (1979) Inhibitory effects of a new oral gold compound on HeLa cells. Cancer 44: 1965–1975. 389401 10.1002/1097-0142(197912)44:6<1965::aid-cncr2820440602>3.0.co;2-6

[19] Mirabelli CK, Johnson RK, Hill DT, Faucette L, Girard GR, et al (1986) Correlation of the *in vitro* cytotoxic and *in vivo* antitumor activities of gold(I) coordination complexes. J Med Chem 29: 218–223. 3081721 10.1021/jm00152a009

[20] Stallings-Mann M, Jamieson L, Regala RP, Weems C, Murray NR, et al (2006) A novel small-molecule inhibitor of protein kinase Ciota blocks transformed growth of non-small-cell lung cancer cells. Cancer Res 66: 1767–1774. 16452237 10.1158/0008-5472.CAN-05-3405

[21] Tiekink ERT (2008) Anti-cancer potential of gold complexes. Inflammopharmacology 16: 138–142. 18521545 10.1007/s10787-007-0018-5

[22] Tiekink ERT (2002) Gold derivatives for the treatment of cancer. Crit Rev Hematol Oncol 42: 225–248. 10.1016/s1040-8428(01)00216-5 12050017

[23] Barreiro E, Casas JS, Couce MD, Sanchez-Gonzalez A, Sordo J, et al (2008) Synthesis, structure and cytotoxicity of triphenylphosphinegold(I) sulfanylpropenoates. J Inorg Biochem 102: 184–192. 17870173 10.1016/j.jinorgbio.2007.07.034

[24] Casas JS, Castellano EE, Couce MD, Ellena J, Sánchez A, et al (2006) A gold(I) complex with a vitamin K3 derivative: characterization and antitumoral activity. J Inorg Biochem 100: 1858–1860. 16965818 10.1016/j.jinorgbio.2006.07.006

[25] Casas JS, Castellano EE, Couce MD, Crespo O, Ellena J, et al (2007) Novel Gold(I) 7-Azacoumarin Complex: Synthesis, Structure, Optical Properties, and Cytotoxic Effects. Inorg Chem 46: 6236–6238. 17602616 10.1021/ic700861a

[26] Ott I, Qian X, Xu Y, Kubutat D, Will J, et al. (2009) A gold(I) phosphine complex containing naphthalimide ligand functions as a TrxR inhibiting antiproliferative agent and angiogenesis inhibitor. J Med Chem 52: , 763–770. 10.1021/jm8012135 19123857

[27] Gallassi R, Burini A, Ricci S, Pellei M, Rigobello MP, et al (2012) Synthesis and characterization of azolate gold(I) phosphane complexes as thioredoxin reductase inhibiting antitumor agents. Dalton Trans 41: 5307–5318. 22391922 10.1039/c2dt11781a

[28] Serratrice M, Cinellu MA, Maiore L, Pilo M, Zucca A, et al (2012) Synthesis, Structural Characterization, Solution Behavior, and *in Vitro* Antiproliferative Properties of a Series of Gold Complexes with 2-(2′-Pyridyl)benzimidazole as Ligand: Comparisons of Gold(III) versus Gold(I) and Mononuclear versus Binuclear Derivatives. Inorg Chem 51: 3161–3171. 22339487 10.1021/ic202639t

[29] Abbehausen C, Peterson EJ, de Paiva RE, Corbi PP, Formiqa AL, et al (2013) Gold(I)-phosphine-*N*-heterocycles: biological activity and specific (ligand) interactions on the C-terminal HIVNCp7 zinc finger. Inorg Chem 52: 11280–11287. 24063530 10.1021/ic401535s

[30] Illán-Cabeza NA, García-García AR, Martínez-Martos JM, Ramírez-Expósito MJ, Pena-Riuz T, et al. (2013) A potential antitumor agent, (6-amino-1-methyl-5-nitrosouracilato-*N*3)-triphenylphosphine-gold(I): Structural studies and *in vivo* biological effects against experimental glioma Eur. J. Med. Chem. 64 , 2013, 260–272. 10.1016/j.ejmech.2013.03.067 23644209

[31] Berners-Price SJ, Mirabelli CK, Johnson RK, Mattern MR, McCabe FL, et al (1986) *In Vivo* Antitumor Activity and *in Vitro* Cytotoxic Properties of Bis[l,2-bis(diphenylphosphino)ethane]gold(I) Chloride. Cancer Res 46: 5486–5493. 3756897

[32] Mirabelli CK, Hill DT, Faucette LF, McCabe FL, Girard GR, et al (1987) Antitumor activity of bis(diphenylphosphino)alkanes, their gold(I) coordination complexes, and related compounds. J Med Chem 30: 2181–2190. 3681888 10.1021/jm00395a004

[33] Berners-Price SJ, Jarrett PS, Sadler PJ, et al (1987) ^31^P NMR Studies of [Au_2_(*μ*-dppe)^2+^)] Antitumor Complexes. Conversion into [Au(dppe)_2_]^+^ Induced by Thiols and Blood Plasma. Inorg Chem 26: 3074–3077.

[34] Berners-Price SJ, Sadler PJ (1988) Phosphine and metal phosphine complexes: Relationship of chemistry to anticancer and other biological activity. Struct Bonding (Berlin) 70: 27–102.

[35] Berners-Price SJ, Girard GR, Hill DT, Sutton BM, Jarrett PS, et al (1990) Cytotoxicity and antitumor activity of some tetrahedral bis(diphosphino)gold(I) chelates. J Med Chem 33: 1386–1392. 2329559 10.1021/jm00167a017

[36] Hickey JL, Ruhayel RA, Barnard PJ, Baker MV, Berners-Price SJ, et al (2008) Mitochondria-targeted chemotherapeutics: the rational design of gold(I) *N*-heterocyclic carbene complexes that are selectively toxic to cancer cells and target protein selenols in preference to thiols. J Am Chem Soc 130: 12570–12571. 18729360 10.1021/ja804027j

[37] Rubbiani R, Kitanovic I, Alborzinia H, Can S, Kitanovic A, et al (2010) Benzimidazol-2-ylidene gold(I) complexes are thioredoxin reductase inhibitors with multiple antitumor properties. J Med Chem 53: 8608–8618. 21082862 10.1021/jm100801e

[38] Trávníček Z, Štarha P, Vančo J, Šilha T, Hošek J, et al (2012) Anti-inflammatory Active Gold(I) Complexes Involving 6-Substituted-Purine Derivatives. J Med Chem 55: 4568–4579. 22541000 10.1021/jm201416p

[39] Bzowska A, Kulikowska E, Shugar D (2000) Purine nucleoside phosphorylase: properties, functions, and clinical aspects. Pharmacol Ther 88: 349–425. 11337031 10.1016/s0163-7258(00)00097-8

[40] Bantia S, Miller PJ, Parker CD, Ananth SL, Horn LL, et al (2001) Purine phosphorylase inhibitor BCX-1777 (Immucillin-H) – a novel potent and orally active immunosuppressive agent. Int Immunopharmacol 1: 1199–1210. 11407314 10.1016/s1567-5769(01)00056-x

[41] Clinch K, Evans GB, Fröhlich RFG, Furneaux RH, Kelly PM, et al (2009) Third-Generation Immucillins: Syntheses and Bioactivities of Acyclic Immucillin Inhibitors of Human Purine Nucleoside Phosphorylase. J Med Chem 52: 1126–1143. 19170524 10.1021/jm801421q PMC2698043

[42] Balakrishnan K, Verma D, O'Brien S, Kilpatrick JM, Chen Y, et al (2010) Phase 2 and pharmacodynamic study of oral forodesine in patients with advanced, fludarabine-treated chronic lymphocytic leukemia. Blood 116: 886–892. 20427701 10.1182/blood-2010-02-272039 PMC2924226

[43] Vrzal R, Štarha P, Dvořák Z, Trávníček Z (2010) Evaluation of in vitro cytotoxicity and hepatotoxicity of platinum(II) and palladium(II) oxalato complexes with adenine derivatives as carrier ligands. J Inorg Biochem 104: 1130–1132. 20673688 10.1016/j.jinorgbio.2010.07.002

[44] Horvat UEI, Dobrzańska L, Strasser CE, Bouwer (neé Potgieter) W, Joone G, et al (2012) Amides of gold(I) diphosphines prepared from N-heterocyclic sources and their *in vitro* and *in vivo* screening for anticancer activity. J Inorg Biochem 111: 80–90. 22498717 10.1016/j.jinorgbio.2012.02.026

[45] Štarha P, Trávníček Z, Popa A, Popa I, Muchová T, et al (2012) Highly in vitro anticancer effective cisplatin derivatives involving halogeno-substituted 7-azaindole. J Inorg Biochem 115: 57–63. 22922312 10.1016/j.jinorgbio.2012.05.006

[46] Gáliková J, Trávníček Z (2014) Effect of different reaction conditions on the structural diversity of zinc(II) complexes with 9-deazahypoxanthine. Polyhedron 79: 269–276.

[47] Zelová H, Hošek J (2013) TNF-alpha signalling and inflammation: interactions between old acquaintances. Inflamm Res 62: 641–651. 23685857 10.1007/s00011-013-0633-0

[48] Dinarello CA (2011) A clinical perspective of IL-1 beta as the gatekeeper of inflammation. Eur J Immunol 41: 1203–1217. 21523780 10.1002/eji.201141550

[49] Sims JE, Smith DE (2010) The IL-1 family: regulators of immunity. Nature Rev Immunol 10: 89–102. 20081871 10.1038/nri2691

[50] Hayden MS, Ghosh S (2008) Shared principles in NF-kappa B signaling. Cell 132: 344–362. 18267068 10.1016/j.cell.2008.01.020

[51] Garber JC, Barbee RW, Bielitzki JT, Clayton LA, Donovan JC, et al.. (2011) Guide for the Care and Use of Laboratory Animals, 8th ed., Washington: The National Academies Press, USA.

[52] Mann FG, Wells AF, Purdie D (1937) The constitution of complex metalic salts: Part IV. The constitution of the phosphine and arsine derivatives of silver and aurous halides. The coordination of the coordinated argentous and aurous complex. J Chem Soc 1828–1836.

[53] Bruce MI, Nicholson BK, Bin Shawkataly O (1989) Synthesis of gold-containing mixed-metal cluster complexes. Inorg Synth 26: 324–328.

[54] Kamath VP, Juarez-Brambila JJ, Morris CB, Winslow CD, Morris PE Jr (2009) Development of a Practical Synthesis of a Purine Nucleoside Phosphorylase Inhibitor: BCX-4208. Org Process Res Dev 13: 928–932.

[55] Gibson AE, Arris CE, Bentley J, Boyle FT, Curtin NJ, et al (2002) Probing the ATP Ribose-Binding Domain of Cyclin-Dependent Kinases 1 and 2 with O6-Substituted Guanine Derivatives. J Med Chem 45: 3381–3393. 12139449 10.1021/jm020056z

[56] Oxford Diffraction, CrysAlis RED and CrysAlis CCD Software (Ver. 1.171.33.52), Oxford Diffraction Ltd., Abingdon, Oxfordshire, UK.

[57] Sheldrick GM (2008) A short history of SHELX. Acta Crystallogr Sect A 64: 112–122. 18156677 10.1107/S0108767307043930

[58] Brandenburg K (2011) DIAMOND, Release 3.2i, Crystal Impact GbR, Bonn, Germany.

[59] Rode HJ (2008) Apoptosis, Cytotoxicity and Cell Proliferation. 4th edition. Mannheim: Roche Diagnostics GmbH., Germany, 178 p.

[60] Livak KJ, Schmittgen TD (2001) Analysis of relative gene expression data using real-time quantitative PCR and the 2(T)(-Delta Delta C) method. Methods 25: 402–408. 11846609 10.1006/meth.2001.1262

[61] Zimmermann M (1983) Ethical guidelines for investigations of experimental pain in conscious animals. Pain 16: 109–110. 6877845 10.1016/0304-3959(83)90201-4

[62] Chang HY, Sheu MJ, Yang CH, Leu ZC, Chang YS, et al.. (2011) Analgesic effects and the mechanisms of anti-inflammation of hispolon in mice. Evid Based Complement Alternat Med (Article ID 478246) DOI: 10.1093/ecam/nep027. 10.1093/ecam/nep027 PMC3136186 19349477

[63] Salemi G, Gueli MC, D'Amelio M, Saia V, Mangiapane P, et al (2009) Blood levels of homocysteine, cysteine, glutathione, folic acid, and vitamin B12 in the acute phase of atherothrombotic stroke. Neurol Sci 30: 361–364.. 19484186 10.1007/s10072-009-0090-2

[64] Nakamoto K (1997) Infrared and Raman Spectra of Inorganic and Coordination Compounds, Part B: Applications in Coordination, Orgametallic and Bioinorganic Chemistry. fifth ed. New York: Wiley.

[65] Faggianhi R, Howard-Locck HE, Lock CJL, Turner MA (1987) The reaction of chloro(triphenylphosphine)gold(I) with 1-methylthymine, Can J Chem. 65: 1568–1575.

[66] Allen FH (2002) The Cambridge Structural Database: a quarter of a million crystal structures and rising. Acta Crystallogr Sect B Struct Sci 58: 380–388. 10.1107/s0108768102003890 12037359

[67] Hošek J, Vančo J, Štarha P, Paraková L, Trávníček Z (2013) Effect of 2-Chloro-Substitution of Adenine Moiety in Mixed-Ligand Gold(I) Triphenylphosphine Complexes on Anti-Inflammatory Activity: The Discrepancy between the In Vivo and In Vitro Models. Plos One 8: e82441. 24312423 10.1371/journal.pone.0082441 PMC3842384

[68] Seitz M, Valbracht J, Quach J, Lotz M (2003) Gold sodium thiomalate and chloroquine inhibit cytokine production in monocytic THP-1 cells through distinct transcriptional and posttranslational mechanisms. Journal of Clinical Immunology 23: 477–484. 15031635 10.1023/b:joci.0000010424.41475.17

[69] Jeon KI, Jeong JY, Jue DM (2000) Thiol-reactive metal compounds inhibit NF-kappa B activation by blocking I kappa B kinase. Journal of Immunology 164: 5981–5989. 10.4049/jimmunol.164.11.5981 10820281

[70] Abdel-Salam OME, Baiuomy AR, El-Shenawy SM, Arbid MS (2003) The anti-inflammatory effects of the phosphodiesterase inhibitor pentoxifylline in the rat. Pharmacol Res 47: 331–340. 12644391 10.1016/s1043-6618(03)00002-1

[71] Shaw CF, Coffer MT, Klingbeil J, Mirabelli CK (1988) Application of phosphorus-31 NMR chemical shift: gold affinity correlation to hemoglobin-gold binding and the first inter-protein gold transfer reaction. J Am Chem Soc 110: 729–734.

[72] Iqbal MS, Taqi SG, Arif M, Wasim M, Sher M (2009) In vitro distribution of gold in serum proteins after incubation of sodium aurothiomalate and auranofin with human blood and its pharmacological significance. Biol Trace Elem Res 130: 204–209. 19194667 10.1007/s12011-009-8330-0

[73] Saccoccia F, Angelucci F, Boumis G, Brunori M, Miele AE, et al (2012) On the mechanism and rate of gold incorporation into thiol-dependent flavoreductases. J Inorg Biochem 108: 105–111. 22166353 10.1016/j.jinorgbio.2011.11.005 PMC3396563

[74] Lewis D, Capell HA, McNeil CJ, Iqbal MS, Brown DH, et al (1983) Gold levels produced by treatment with auranofin and sodium aurothiomalate. Ann Rheum Dis 42: 566–570. 6414387 10.1136/ard.42.5.566 PMC1001298

