# Supplementary material for: Gold(I) Complexes of 9-Deazahypoxanthine as Selective Antitumor and Anti-Inflammatory Agents
Source: PLoS One. 2014 Oct 15;9(10):e109901. doi: 10.1371/journal.pone.0109901 (PMC4198181; doi:10.1371/journal.pone.0109901)
Supplement: Information S1 — Synthesis, elemental analysis and ESI MS, FT-IR, 1H and 13C NMR data for HL1–5 as well as the results of elemental analysis, TG/DTA, ESI MS, FT-IR, 1H and 13C NMR experiments assigned to complexes 1–5 are given in Information S1. Figure S1. TG/DTA curves of the complexes 2 and 4. Figure S2. ESI+ MS spectrum of 4. Figure S3. 1H and 13C NMR spectra of 2. Figure S4. 1H–13C HMQC NMR spectra of 2. Figure S5. A part of the crystal structure of HL5. Table S1. Crystal data and structure refinements for HL5 and 2. Table S2. Selected bond lengths and angles in HL5. Table S3. Selected bond lengths and angles in complex 2. Table S4. Selected non-covalent contacts in the crystal structure of HL5. Table S5. Selected non-covalent contacts in the crystal structure complex 2. (DOCX) [file pone.0109901.s001.docx]

**SUPPORTING INFORMATION**

**Gold(I) Complexes of 9-Deazahypoxanthine as Selective Antitumor and Anti-Inflammatory Agents**

Ján Vančo^1^, Jana Gáliková^1^, Jan Hošek^1^, Zdeněk Dvořák^2^, Lenka Paráková^3^, Zdeněk Trávníček^1^*

*^1^ Regional Centre of Advanced Technologies and Materials & Department of Inorganic Chemistry, Faculty of Science, Palacký University, Olomouc, Czech Republic*

*^2^ Regional Centre of Advanced Technologies and Materials & Department of Cell Biology and Genetics, Faculty of Science, Palacký University, Olomouc, Czech Republic*

*^3^ Department of Human Pharmacology and Toxicology, Faculty of Pharmacy, University of Veterinary and Pharmaceutical Sciences Brno, Brno, Czech Republic*

E-mail addresses: JV ([jan.vanco@upol.cz](mailto:jan.vanco@upol.cz)), JG ([jana.galikova@upol.cz](mailto:jana.galikova@upol.cz)), JH ([jan.hosek@upol.cz](mailto:jan.hosek@upol.cz)), ZD ([zdenek.dvorak@upol.cz](mailto:zdenek.dvorak@upol.cz)), LP ([parakoval@vfu.cz](mailto:parakoval@vfu.cz)), ZT ([zdenek.travnicek@upol.cz](mailto:zdenek.travnicek@upol.cz))

* Corresponding author: Zdeněk Trávníček, Regional Centre of Advanced Technologies and Materials & Department of Inorganic Chemistry, Faculty of Science, Palacký University, 17. listopadu 12, CZ-771 46 Olomouc, Czech Republic; Tel.: +420-585-634-352; fax: +420-585-634-954; e-mail: zdenek.travnicek@upol.cz**Contents:**

**Synthesis and characterization of the ligands HL_1_–HL_2_................................... Pages S3-S4**

**Synthesis and characterization of the ligands HL_3_–HL_5_............................…….. Pages S5-S7**

**Elemental analysis, FT-IR, NMR and ESI–MS data for complexes 1-5.............. PagesS8-S12**

**Figure S1. TG/DTA curves of the complexes 2 and 4....................................... Page S13**

**Figure S2. ESI+ MS spectrum of 4................................................................... Page S14**

**Figure S3. ^1^H and ^13^C NMR spectra of 2.......................................................... Page S15**

**Figure S4. ^1^H–^13^C HMQC NMR spectra of 2..................................................... Page S16**

**Table S1. Crystal data and structure refinements for HL_5_ and 2...................... Page S17**

**Table S2. Selected bond lengths and angles for HL_5_........................................ Page S18**

**Table S3. Selected bond lengths and angles for complex 2............................. Page S19**

**Table S4. Selected non-covalent contacts in the crystal structure of HL_5_........ Page S20**

**Table S5. Selected non-covalent contacts in the crystal structure of 2............ Page S21**

**Figures S5. A part of the crystal structure of HL_5_............................................. Page S22**

**Synthesis and characterization of the ligands HL_1_–HL_2_**

The appropriate alcohol (i.e. 14 mL of dry ethanol, or 20 ml of dry isopropanol) and sodium (15 mmol) were stirred under a nitrogen atmosphere. Then, 6-chloro-9-deazapurine (6.5 mmol) was added, and stirring was continued under reflux for 24 hours. The reaction mixture was concentrated *in vacuo* and the residues were suspended in 10 mL of water and then neutralized to pH 6.5–7.5 with addition of 0.1 M solution of hydrochloric acid. The neutralized solution was extracted with ethyl acetate (3 × 10 mL). The combined organic extracts were dried (MgSO_4_) and the solvent was evaporated *in vacuo* to isolate the product. The obtained product was filtered off, washed with diethyl ether (5 mL) and recrystallized from ethanol.

**HL_1_:** Yield: 47%. Mp.: 150 °C. *Anal*. Calc. for C_8_H_9_N_3_O (M_r_ = 163.2): C, 58.9%; H, 5.6%; N, 25.8%. Found: C, 58.8%; H, 5.8%; N, 25.5%. ESI+ mass spectra (methanol, *m/z*) 164.0 (calc. 164.1) [HL_1_+H]^+^. FT-IR (ν_ATR_/cm^–1^): 3236w ν(N–H)_ar_; 3140m, 3054m ν(C–H)_ar_; 2987s ν(C–H)_aliph_; 2907s; 2800s; 2754s; 2699s; 2635s; 2528m; 1624vs ν(C^…^N)_ring_; 1543s, 1500s, 1479m ν(C^…^C)_ring_; 1441s; 1410m; 1374s; 1334vs ν(C_6_–O); 1295s; 1263m; 1198m; 1137m; 1097vs ν(O–C_10_); 1064m; 1017m; 945w; 905s; 893s; 790s; 752w; 724m; 665m; 640m; 593s; 514w. ^1^H NMR, DMF-*d_7_*, SiMe_4_, δ, ppm: 12.06 (bs, N7H, 1H), 8.42 (s, C2H, 1H), 7.74 (m, C8H, 1H), 6.58 (m, C9H, 1H), 4.59 (q, 7, C10H, 2H), 1.43 (t, 7, C11H, 3H). ^13^C NMR, DMF-*d_7_*, SiMe_4_, δ, ppm: 155.84 (C6), 151.09 (C4), 149.59 (C2), 130.37 (C8), 115.23 (C5), 102.11 (C9), 61.89 (C10), 14.43 (C11).


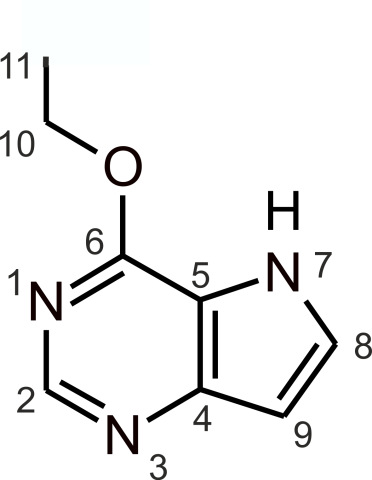


**HL_2_:** Yield: 43%. Mp.: 150 °C. *Anal*. Calc. for C_9_H_11_N_3_O (M_r_ = 177.2): C, 61.0%; H, 6.3%; N, 23.7%. Found: C, 61.1%; H, 6.5%; N, 22.3%. ESI+ mass spectra (methanol, *m/z*): 178.0 (calc. 178.1) [HL_2_+H]^+^. FT–IR (ν_ATR_/cm^–1^): 3240w ν(N–H)_ar_; 3136m, 3085s, 3038m ν(C–H)_ar_; 2986vs ν(C–H)_aliph_; 2938s; 2813s; 2753s; 2695s; 2637m; 2523m; 1619s ν(C^…^N)_ring_; 1540s, 1503s, 1491s ν(C^…^C)_ring_; 1468s; 1438s; 1400s; 1372vs; 1321s ν(C_6_–O); 1297s; 1263m; 1204m; 1172w; 1136m; 1104s, 1090s ν(O–C_10_); 1055m; 958w; 914m; 895s; 813m; 793s; 737m; 670w; 642m; 595m; 520w; 460w. ^1^H NMR, DMF-*d_7_*, SiMe_4_, δ, ppm: 12.06 (bs, N7H, 1H), 8.43 (s, C2H, 1H), 7.73 (m, C8H, 1H), 6.57 (m, C9H, 1H), 5.59 (sept, 6.3, C10H, 1H), 1.41 (d, 6.3, C11H, C12H, 6H). ^13^C NMR, DMF-*d_7_*, SiMe_4_, δ, ppm: 155.54 (C6), 151.10 (C4), 149.60 (C2), 130.27 (C8), 115.50 (C5), 102.08 (C9), 68.91 (C10), 21.85 (C11, C12).


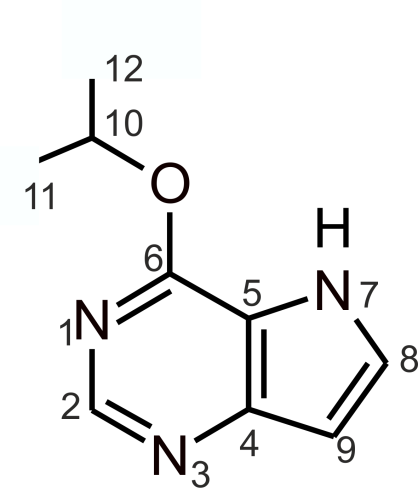


**Synthesis and characterization of the ligands HL_3_–HL_5_**

The appropriate alcohol (40 mmol) and sodium hydride (8 mmol) were stirred under nitrogen atmosphere. Then, 6-chloro-9-deazapurine (4 mmol) was added, and stirring continued under reflux for the next 5 days. The reaction mixture was cooled to laboratory temperature, suspended in water (10 mL) and extracted with ethyl acetate (3 × 10 mL). The combined organic extracts were dried (MgSO_4_) and the solvent was evaporated *in vacuo* to isolate the product. The white product was filtered off, washed with diethyl ether (5 mL) and recrystallized from acetonitrile.

**HL_3_:** Yield: 47%. Mp.: 101 °C. *Anal*. Calc. for C_11_H_13_N_3_O_2_ (M_r_ = 219.2): C, 60.3%; H, 6.0%; N, 19.2%. Found: C, 60.1%; H, 6.2%; N, 18.9%. ESI+ mass spectra (methanol, *m/z*): 206.1 (calc. 206.1) [HL_3_ +H]^+^. FT–IR (ν_ATR_/cm^–1^): 3236w ν(N–H)_ar_; 3120s; 3085s; 3036s ν(C–H)_ar_; 2966s; 2940s; 2875s; 2848vs; 1619vs ν(C^…^N)_ring_; 1537s; 1497s; 1485s ν(C^…^C)_ring_; 1464w; 1435s; 1410m; 1392m; 1377s; 1357m; 1338s, 1324s ν(C_6_–O); 1302s; 1260m; 1218w; 1189m; 1119m; 1095s, 1083s ν(O–C_10_); 1064s; 1020s; 961w; 948m; 931w; 884s; 845m; 798s; 755m; 670w; 654m; 620w; 589m; 548w; 553w; 477w. ^1^H NMR, DMF-*d_7_*, SiMe_4_, δ, ppm: 12.27 (bs, N7H, 1H), 8.44 (s, C2H, 1H), 7.76 (m, C8H, 1H), 6.60 (m, C9H, 1H), 4.57–4.50 (m, C10H, 2H), 4.29 (qd, 6.8, C11H, 1H), 3.86–3.76 (m, C14H, 2H), 2.06–1.77 (m, C12H, C13H, 4H). ^13^C NMR, DMF-*d_7_*, SiMe_4_, δ, ppm: 155.83 (C6), 151.22 (C4), 149.45 (C2), 130.55 (C8), 115.12 (C5), 102.13 (C9), 76.96 (C11), 68.21, 68.17 (C10, C14), 28.10, 25.84 (C12, C13).


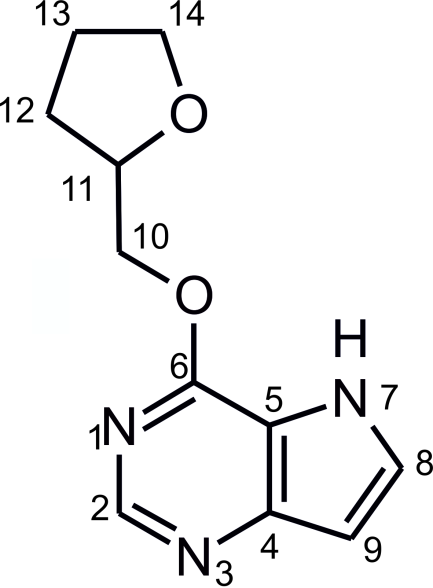


**HL_4_:** Yield: 34%. Mp.: 154 °C. *Anal*. Calc. for C_13_H_11_N_3_O (M_r_ = 225.2): C, 69.3%; H, 4.9%; N, 18.7%. Found: C, 69.4%; H, 5.1%; N, 18.5%. ESI+ mass spectra (methanol, *m/z*): 226.4 (calc. 226.1) [HL_4_+H]^+^. FT–IR (ν_ATR_/cm^–1^): 3406w ν(N–H)_ar_; 3139m, 3049s, 3027s ν(C–H)_ar_; 3005s; 2960s; 2829s; 2794s; 2747s; 2692s; 1624vs ν(C^…^N)_ring_; 1535s, 1493s ν(C^…^C)_ring_; 1452s; 1440s; 1403s; 1377m; 1362m; 1327vs ν(C_6_–O); 1295s; 1266m; 1242m; 1208m; 1189m; 1127m; 1093s ν(O–C_10_); 1079m; 1027s; 973w; 933m; 895s; 820m; 795m; 740m; 694s; 664w; 646m; 637m; 593m; 521w. ^1^H NMR, DMF-*d_7_*, SiMe_4_, δ, ppm: 12.23 (bs, N7H, 1H), 8.48 (s, C2H, 1H), 7.78 (m, C8H, 1H), 7.59–7.45 (m, C12H, C13H, C14H. C15H, C16H, 5H), 6.61 (m, C9H, 1H), 5.66 (s, C10H, 2H). ^13^C NMR, DMF-*d_7_*, SiMe_4_, δ, ppm: 155.63 (C6), 151.32 (C4), 149.46 (C2), 137.44 (C11), 130.65 (C8), 128.78 (C13, C15), 128.38 (C14), 128.30 (C12, C16), 115.16 (C5), 102.18 (C9), 67.40 (C10).


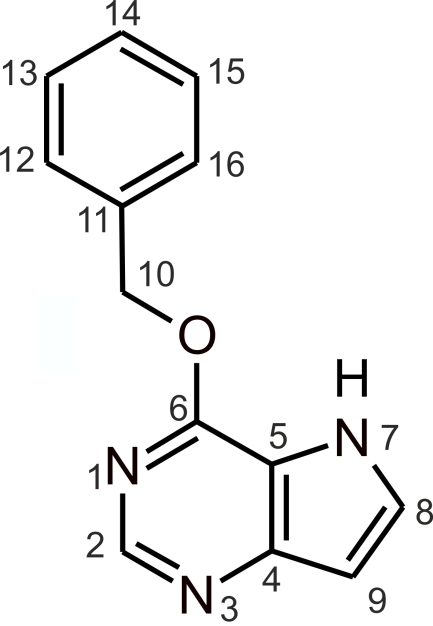


**HL_5_:** Yield: 41%. Mp.: 121 °C. *Anal*. Calc. for C_14_H_13_N_3_O (M_r_ = 239.3): C, 70.3%; H, 5.5%; N, 17.6%. Found: C, 70.0%; H, 5.4%; N, 17.5%. ESI+ mass spectra (methanol, *m/z*): 240.2 (calc. 240.1) [HL_5_+H]^+^. FT-IR (ν_ATR_/cm^–1^): 3244w ν(N–H)_ar_; 3138w, 3083m, 3060m, 3045m, 3023m ν(C–H)_ar_; 2963m; 2923m; 2840m; 2797m; 2753m; 2697m; 2632m; 1627vs ν(C^…^N)_ring_; 1535s, 1493s, 1489s ν(C^…^C)_ring_; 1452s; 1437m; 1408m; 1375m; 1335vs ν(C_6_–O); 1301m; 1288w; 1262w; 1214w; 1187w; 1131m; 1100vs ν(O–C_10_); 1054w; 1029w; 987w; 961w; 892s; 840w; 794m; 771w; 748s; 726w; 699m; 668w; 647m; 596m; 513w; 492w. ^1^H NMR, DMF-*d_7_*, SiMe_4_, δ, ppm: 12.14 (bs, N7H, 1H), 8.45 (s, C2H, 1H), 7.76 (m, C8H, 1H), 7.41 (m, C14H, C16H, 2H), 7.33 (m, C13H, C17H, 2H), 7.27 (m, C15H, 1H), 6.59 (m, C9H, 1H), 4.76 (m, C10H, 2H), 3.18 (t, 6.8, C11H, 2H). ^13^C NMR, DMF-*d_7_*, SiMe_4_, δ, ppm: 155.78 (C6), 150.97 (C4), 149.49 (C2), 138.90 (C12), 130.59 (C8), 129.42 (C14, C16), 128.69 (C13, C17), 126.67 (C15), 115.15 (C5), 102.06 (C9), 66.89 (C10), 35.29 (C11).


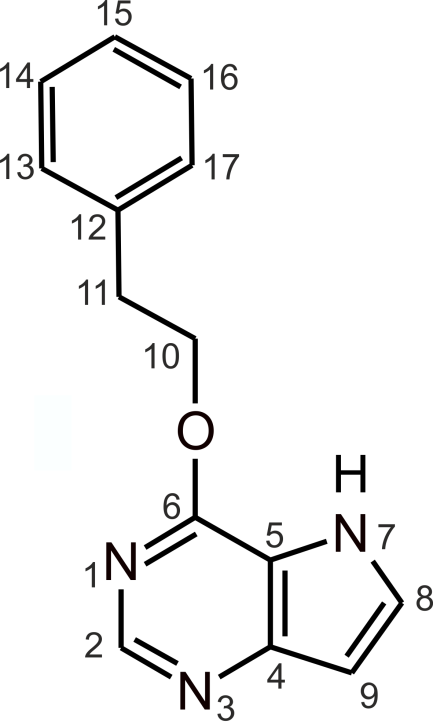


**Elemental analysis, FTIR, NMR and ESI–MS data for complexes 1-5**

[Au(L_1_)(PPh_3_)] (**1**): Yield: 63% [with respect to Au]. *Anal*. Calc. for C_26_H_23_N_3_OPAu (M_r_ = 621.4): C, 50.2%; H, 3.7%; N, 6.8 %. Found: C, 50.3%; H, 3.8%; N, 6.4%. TG/DTA data: decomposition began at 71 °C and finished at 733 °C with a weight loss of 67.6% (calc. to Au residue: 68.3%), endothermic peaks at 207 and 283 °C. ESI+ mass spectra (methanol, *m/z*): 622.2 (calc. 622.1) [Au(L_1_)(PPh_3_)+H]^+^; 644.1 (calc. 644.1) [Au(L_1_)(PPh_3_)+Na]^+^. FT-IR (ν_ATR_/cm^–1^): 3072w, 3047w, 3023w ν(C–H)_ar_; 2975m ν(C–H)_aliph_; 2925w; 2856w; 1589vs ν(C^…^N)_ring_; 1545w, 1522m, 1502w, 1479w ν(C^…^C)_ring_; 1453m; 1435s; 1400m; 1372m; 1332s ν(C_6_–O); 1281m; 1189w; 1163m; 1101vs ν(O–C_10_); 1015w; 997w; 951w; 896w; 793w; 749m; 692s; 671w; 622w, 596w; 545vs; 535m; 509s ν(Au–N); 502s; 448w; 436w; 340w ν(Au–P). ^1^H NMR, DMF-*d_7_*, SiMe_4_, δ, ppm: 8.28 (s, C2H, 1H), 7.78 (m, C21H, C25H, C31H, C35H, C41H, C45H, 6H, PPh_3_), 7.71 (m, C22H, C23H, C24H, C32H, C33H, C34H, C42H, C43H, C44H, 9H, PPh_3_), 7.64 (m, C8H, 1H), 6.54 (m, C9H, 1H), 4.54 (q, 6.8, C10H, 2H), 1.17 (t, 6.8, C11H, 3H). ^13^C NMR, DMF-*d_7_*, SiMe_4_, δ, ppm: 156.66 (C6), 152.16 (C4), 147.42 (C2), 140.47 (C8), 134.67, 134.53 (C21, C25, C31, C35, C41, C45, PPh_3_), 132.65, 132.67 (C22, C24, C32, C34, C42, C44, PPh_3_), 130.06, 129.94 (C23, C33, C43, PPh_3_), 129.75 (C20, C30, C40, PPh_3_), 122.67 (C5), 101.32 (C9), 61.43 (C10), 14.68 (C11).


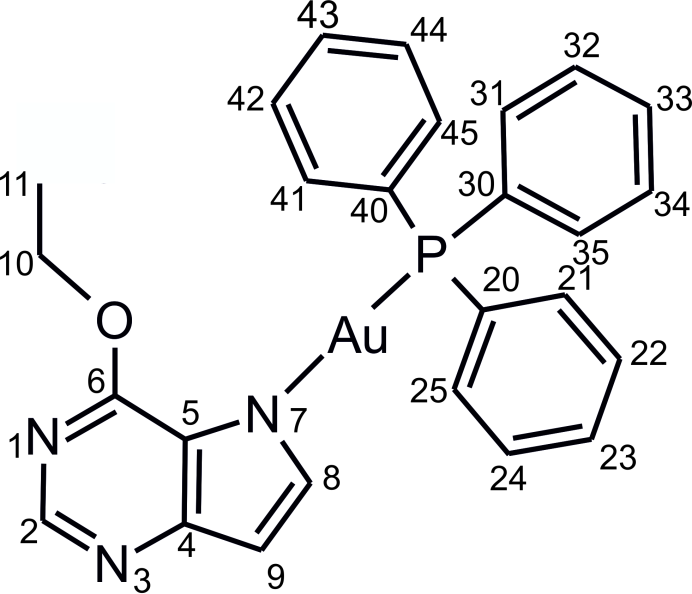


[Au(L_2_)(PPh_3_)] (**2**): Yield: 69% [with respect to Au]. *Anal*. Calc. for C_27_H_25_N_3_OPAu (M_r_ = 635.4): C, 51.0%; H, 4.0%; N, 6.6%. Found: C, 50.9%; H, 4.0%; N, 6.4%. TG/DTA data: decomposition began at 160 °C and finished at 729 °C with a weight loss of 68.1% (calc. to Au residue: 69.1%), endothermic peaks at 236 and 266 °C and exothermic peaks at 467 and 599 °C. ESI+ mass spectra (methanol, *m/z*): 636.2 (calc. 636.2) [Au(L_2_)(PPh_3_)+H]^+^; 658.1 (calc. 658.1) [Au(L_2_)(PPh_3_)+Na]^+^. FT-IR (ν_ATR_/cm^–1^): 3053w, 3030w ν(C–H)_ar_; 2974m ν(C–H)_aliph_; 2926w; 2867w; 1591vs ν(C^…^N)_ring_; 1517s, 1499w, 1478m ν(C^…^C)_ring_; 1452s; 1436s; 1399s; 1365s; 1324s ν(C_6_–O); 1281s; 1200w; 1164s; 1139w; 1098vs ν(O–C_10_); 1026w; 996w; 949w; 918m; 876m; 819w; 792w; 751s; 710m; 692s; 671m; 651w; 620m, 574w; 544vs; 507vs ν(Au–N); 451w; 434w; 295w ν(Au–P). ^1^H NMR, DMF-*d_7_*, SiMe_4_, δ, ppm: 8.27 (s, C2H, 1H), 7.80 (m, C21H, C25H, C31H, C35H, C41H, C45H, 6H, PPh_3_), 7.71 (m, C22H, C23H, C24H, C32H, C33H, C34H, C42H, C43H, C44H, 9H, PPh_3_), 7.64 (d, 2.3, C8H, 1H), 6.54 (d, 2.3, C9H, 1H), 5.56 (m, C10H, 1H), 1.15 (d, 5.9, C11H, C12H, 6H). ^13^C NMR, DMF-*d_7_*, SiMe_4_, δ, ppm: 156.40 (C6), 152.17 (C4), 147.45 (C2), 140.36 (C8), 134.73, 134.60 (C21, C25, C31, C35, C41, C45, PPh_3_), 132.67, 132.65 (C22, C24, C32, C34, C42, C44, PPh_3_), 130.08, 129.96 (C23, C33, C43, PPh_3_), 129.76 (C20, C30, C40, PPh_3_), 123.14 (C5), 101.32 (C9), 68.06 (C10), 22.01 (C11, C12).


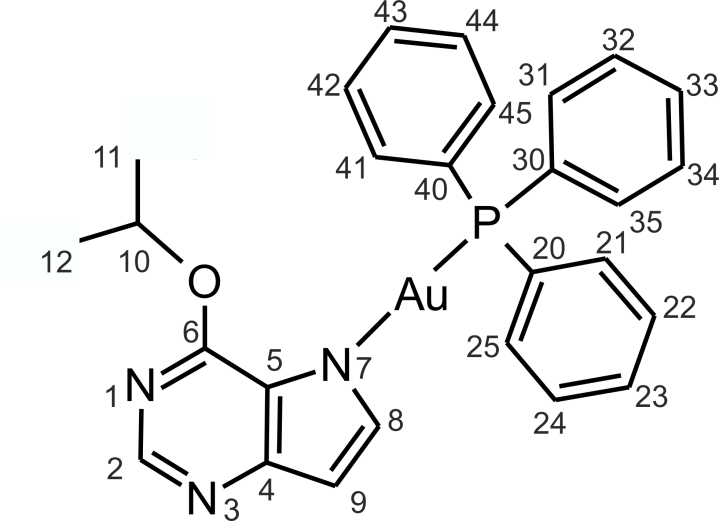


[Au(L_3_)(PPh_3_)] (**3**): Yield: 75% [with respect to Au]. *Anal*. Calc. for C_29_H_27_N_3_O_2_PAu (M_r_ = 677.5) C, 51.4%; H, 4.0%; N, 6.2%. Found: C, 51.3%; H, 4.0%; N, 5.9%. TG/DTA data: decomposition began at 169 °C and finished at 714 °C with a weight loss of 66.1% (calc. to Au residue: 70.5%) and endothermic peaks at 202 and 286 °C and exothermic peaks at 512 °C. ESI+ mass spectra (methanol, *m/z*): 678.1 (calc. 678.1) [Au(L_3_)(PPh_3_)+H]^+^, 700.2 (calc. 700.1) [Au(L_3_)(PPh_3_)+Na]^+^. FT-IR (ν_ATR_/cm^–1^): 3070w, 3046w, 3018w ν(C–H)_ar_; 2969w; 2949w; 2865w; 1590vs ν(C^…^N)_ring_; 1519m, 1479w, 1470w ν(C^…^C)_ring_; 1456m; 1435s; 1398s; 1369s; 1328m ν(C_6_–O); 1278s; 1241w; 1183m; 1161s; 1101vs ν(O–C_10_); 1069m; 1025w; 996w; 967w; 950w; 930w; 869w; 792m; 748s; 710m; 692s; 669m; 649w; 619m, 595w; 545vs; 509s ν(Au–N); 499s; 452w; 431w; 391w; 330w; 297w ν(Au–P); 276w. ^1^H NMR, DMF-*d_7_*, SiMe_4_, δ, ppm: 8.28 (bs, C2H, 1H), 7.79 (m, C21H, C25H, C31H, C35H, C41H, C45H, 6H, PPh_3_), 7.71 (m, C22H, C23H, C24H, C32H, C33H, C34H, C42H, C43H, C44H, 9H, PPh_3_), 7.66 (d, 2, C8H, 1H), 6.55 (m, C9H, 1H), 4.52–4.44 (m, C10H, 2H), 3.91 (m, C11H, 1H), 3.60–3.36 (m, C14H, 2H), 1.67–1.54 (m, C12H, C13H, 4H). ^13^C NMR, DMF-*d_7_*, SiMe_4_, δ, ppm: 156.60 (C6), 152.26 (C4), 147.29 (C2), 140.71 (C8), 134.77, 134.64 (C21, C25, C31, C35, C41, C45, PPh_3_), 132.60 (C22, C24, C32, C34, C42, C44, PPh_3_), 130.01, 129.90 (C23, C33, C43, PPh_3_), 129.72 (C20, C30, C40, PPh_3_), 122.70 (C5), 101.35 (C9), 76.96, 76.90 (C11), 67.82, 67.77 (C14, C10), 28.45, 25.49 (C12, C13).


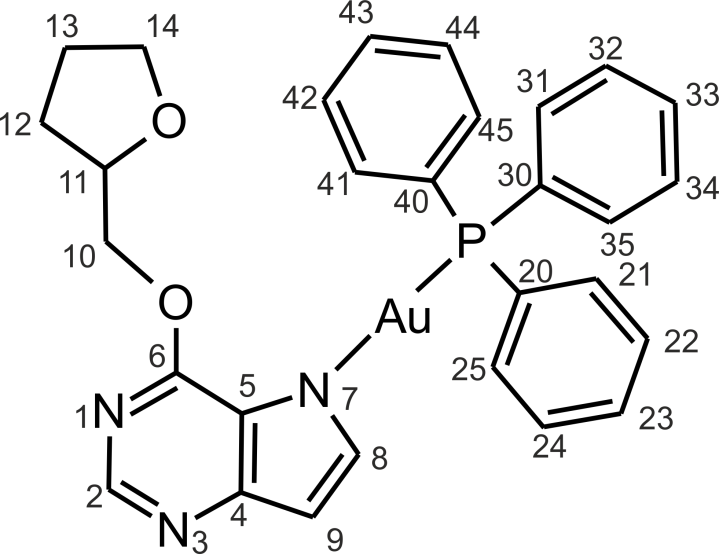


[Au(L_4_)(PPh_3_)] (**4**): Yield: 65% [with respect to Au]. *Anal*. Calc. for C_31_H_25_N_3_OPAu (M_r_ = 683.5) C, 54.5%; H, 3.7%; N, 6.2%. Found: C, 54.5%; H, 3.8%; N, 5.9%. TG/DTA data: decomposition began at 125 °C and finished at 759 °C with a weight loss of 69.7% (calc. to Au residue: 70.7%) and endothermic peak at 198 °C. ESI+ mass spectra (methanol, *m/z*): 684.2 (calc. 684.2) [Au(L_4_)(PPh_3_)+H]^+^, 706.2 (calc. 706.1) [Au(L_4_)(PPh_3_)+ Na]^+^. FT-IR (ν_ATR_/cm^–1^): 3077w, 3052w, 3022w ν(C–H)_ar_; 2968w, 2931w, 2849w, 1593vs ν(C–N)_ring_; 1522s, 1494w, 1476m ν(C^…^C)_ring_; 1454m, 1434s, 1392m, 1365m, 1330m ν(O–C_6_); 1309w, 1295w, 1278m, 1254w, 1187w, 1159m, 1100vs ν(O–C_10_); 1071w, 1055w, 1026w, 996w, 973w, 947w, 901w, 866w, 848w, 792w, 741s, 710m; 690vs, 667m, 622w, 602w, 570w, 544vs, 504s ν(Au–N), 445w, 432w, 338w, 310w ν(Au–P), 259w. ^1^H NMR, DMF-*d_7_*, SiMe_4_, δ, ppm: 8.32 (s, C2H, 1H), 7.68 (m, C21H, C25H, C31H, C35H, C41H, C45H, 6H, PPh_3_, C8H, 1H), 7.64 (m, C22H, C23H, C24H, C32H, C33H, C34H, C42H, C43H, C44H, 9H, PPh_3_), 7.4 (d, 7.5, C12H, C16H, 2H), 7.1 (m, C14H, 1H), 7.01 (m, C13H, C15H, 2H), 6.59 (d, 2,2, C9H, 1H), 5.68 (s, C10H, 2H). ^13^C NMR, DMF-*d_7_*, SiMe_4_, δ, ppm: 156.37 (C6), 152.46 (C4), 147.25 (C2), 140.84 (C8), 138.07 (C11), 134.63, 134.50 (C21, C25, C31, C35, C41, C45, PPh_3_), 132.56, 132.54 (C22, C24, C32, C34, C42, C44, PPh_3_), 130.00, 129.88 (C23, C33, C43, PPh_3_), 129.61 (C20, C30, C40, PPh_3_), 128.99 (C11), 128.57 (C13, C15), 127.92 (C12, C16), 127.83 (C14), 122.81 (C5), 101.46 (C9), 66.69 (C10).


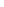

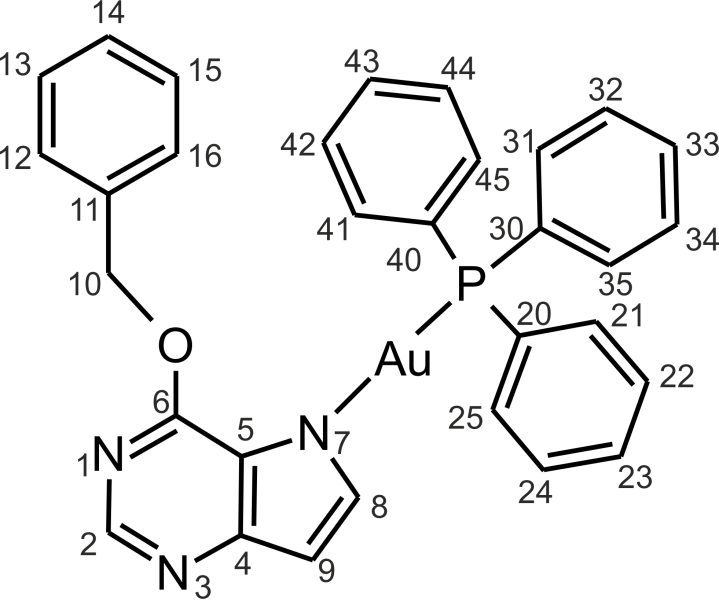


[Au(L_5_)(PPh_3_)] (**5**): Yield: 70% [with respect to Au]. *Anal*. Calc. for C_32_H_27_N_3_OPAu (M_r_ = 697.5) C, 55.1%; H, 3.9%; N, 6.0%. Found: C, 55.3%; H, 3.9%; N, 6.0%. TG/DTA data: decomposition began at 146 °C and finished at 683 °C with a weight loss of 71.9% (calc. to Au residue: 71.3%), endothermic peak at 194 and 319 °C. ESI+ mass spectra (methanol, *m/z*): 698.1 (calc. 698.2) [Au(L_5_)(PPh_3_)+H]^+^, 720.2 (calc. 720.1) [Au(L_5_)(PPh_3_)+Na]^+^. FT-IR (ν_ATR_/cm^–1^): 3063w; 3028w ν(C–H)_ar_; 2960w; 2883w; 1591vs ν(C^…^N)_ring_; 5119m; 1497w; 1478w ν(C^…^C)_ring_; 1454s; 1435m; 1397m; 1368m; 1330s ν(C_6_–O); 1281m; 1191w; 1165m; 1100vs ν(O–C_10_); 1025w; 995w; 965w; 930w; 877w; 848w; 791w; 742m; 711w; 621w; 598w, 561w, 545vs, 506s ν(Au–N), 435w, 391s, 289w ν(Au–P), 261w. ^1^H NMR, DMF-*d_7_*, SiMe_4_, δ, ppm: 8.31 (s, C2H, 1H), 7.75 (m, C21H, C25H, C31H, C35H, C41H, C45H, 6H, PPh_3_), 7.70 (m, C22H, C23H, C24H, C32H, C33H, C34H, C42H, C43H, C44H, 9H, PPh_3_), 7.68 (m, C8H, 1H), 7.17 (m, C13H, C14H, C16H, C17H, 4H), 7.07 (m, C15H, 1H), 6.58 (d, 1.6, C9H, 1H), 4.72 (t, 7.2, C10H, 2H), 2.89 (m, C11H, 2H). ^13^C NMR, DMF-*d_7_*, SiMe_4_, δ, ppm: 156.49 (C6), 152.33 (C4), 147.29 (C2), 140.72 (C8), 138.73 (C12), 134.70, 134.56 (C21, C25, C31, C35, C41, C45, PPh_3_), 132.64, 132.62 (C22, C24, C32, C34, C42, C44, PPh_3_), 130.05, 129.94 (C23, C33, C43, PPh_3_), 129.67 (C20, C30, C40, PPh_3_), 129.24, 129.05, 128.64 (C13, C14, C16, C17), 126.54 (C15), 122.80 (C5), 101.39 (C9), 66.31 (C10), 35.42 (C11).


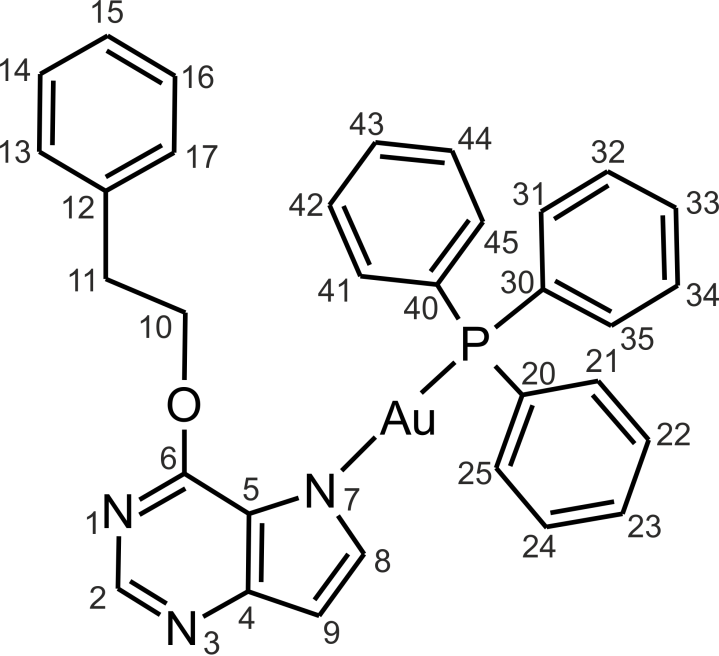

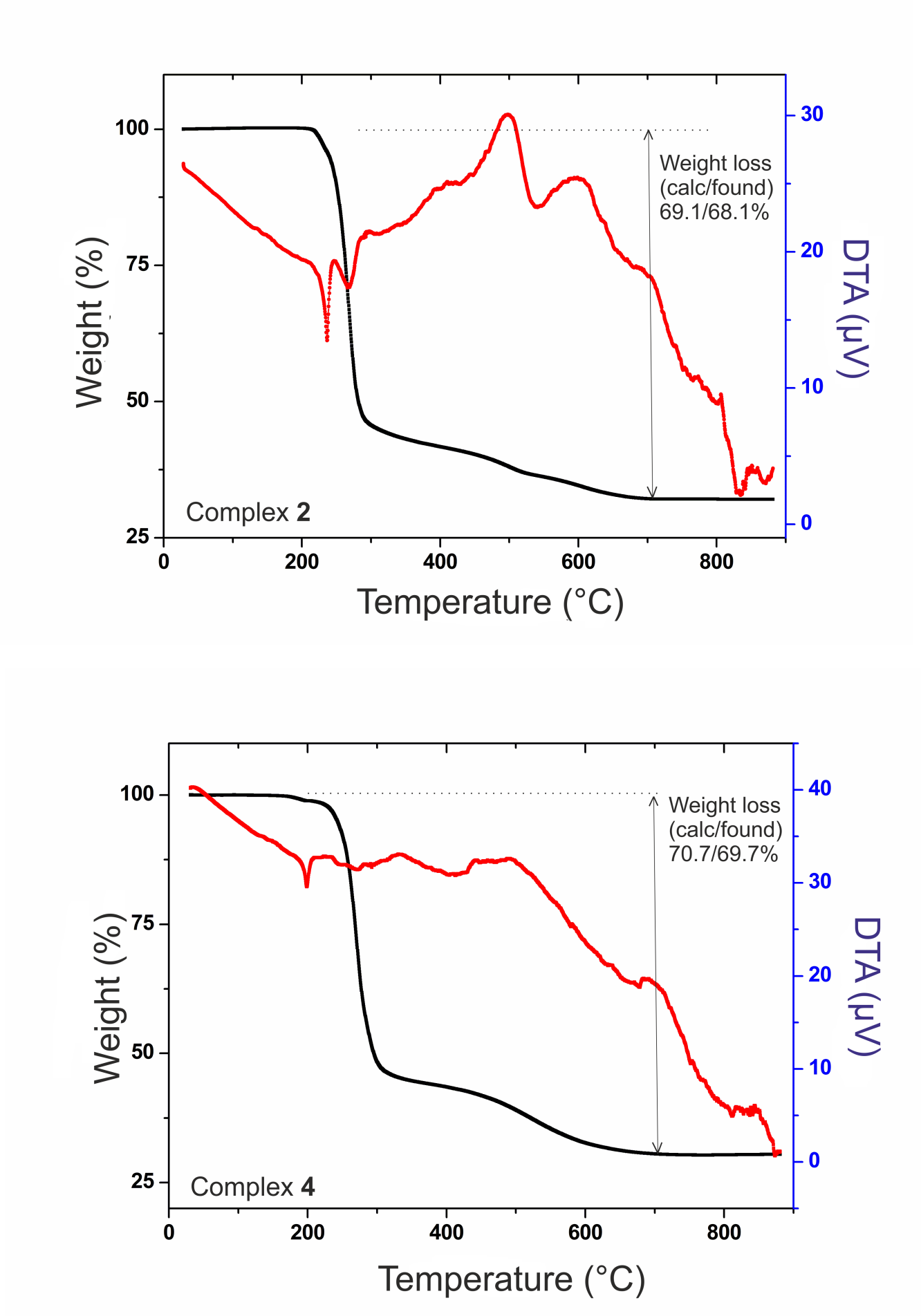


**Figure S1.** The results of simultaneous TG/DTA thermal analysis of the complexes **2** (*up*) and **4** (*down*) showing the TG and DTA curves and observed and calculated weight losses for the final products of the decompositions (determined as a pure gold).


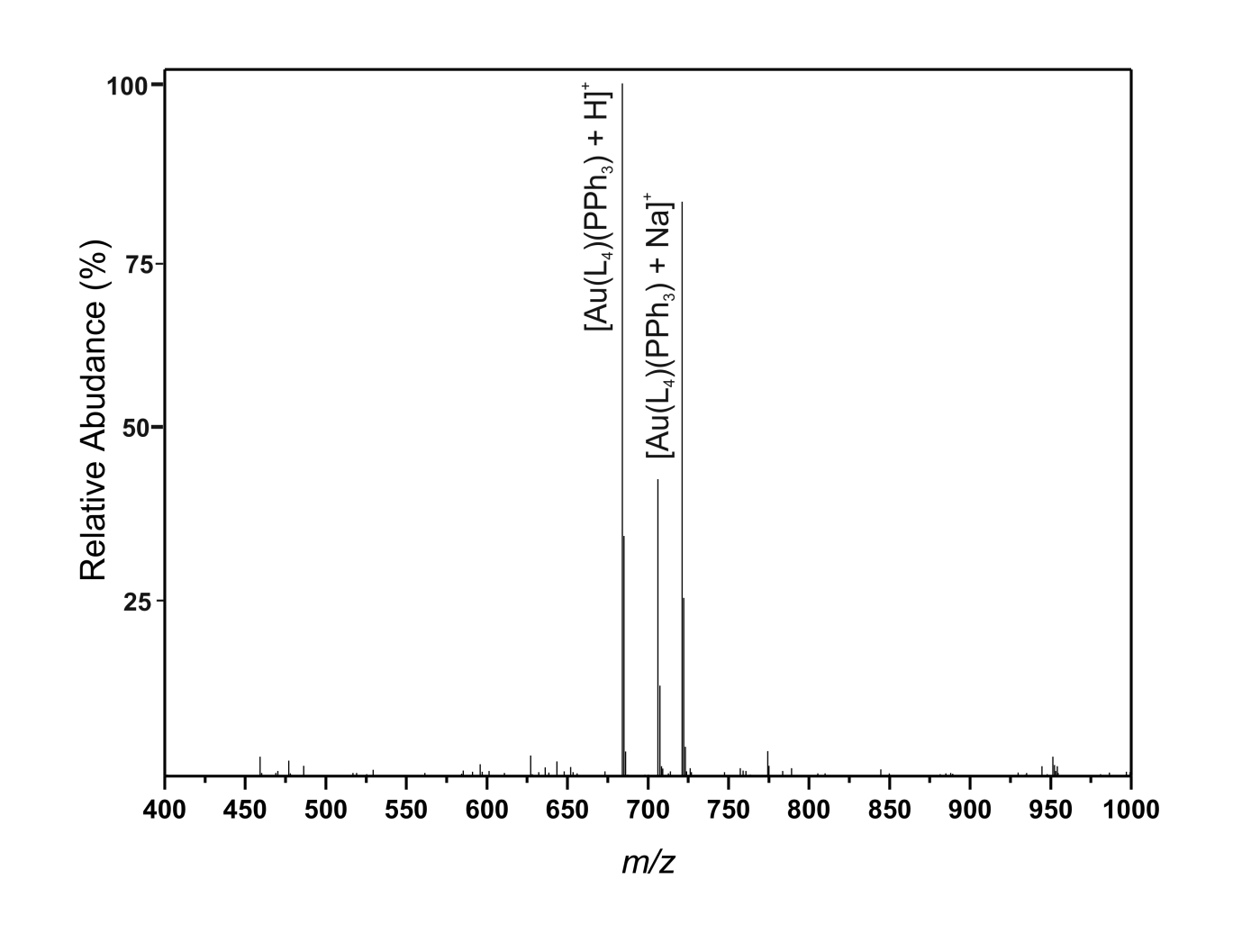


**Figure S2.** ESI+ mass spectrum of **4** dissolved in methanol.


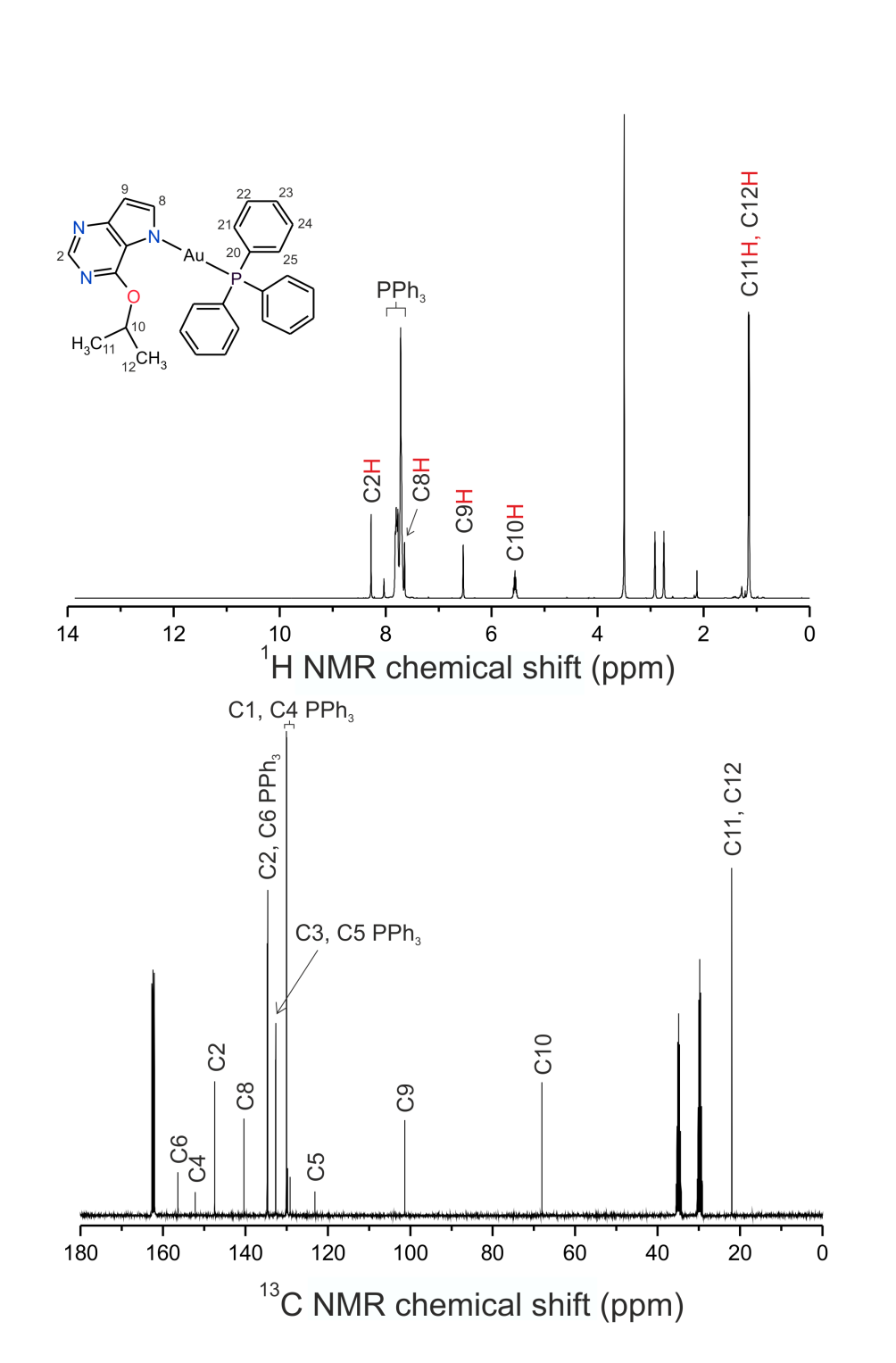


**Figure S3.** ^1^H and ^13^C NMR spectra of **2**.


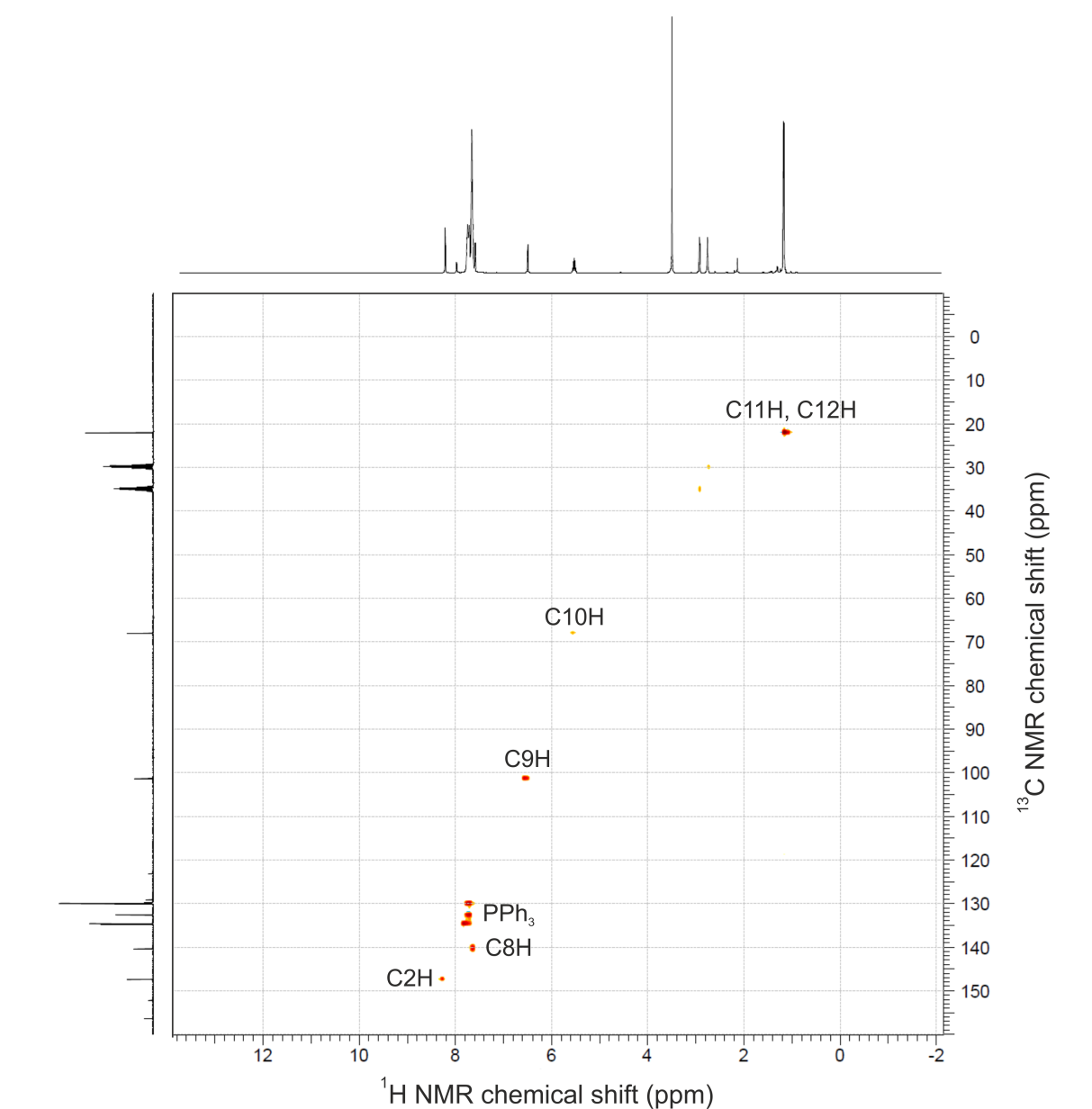


**Figure S4.** ^1^H–^13^C HMQC NMR spectra of **2**.

**Table S1.** Crystallographic data for 6-phenethyloxy-9-deazapurine (HL_5_) and complex **2**.

| *Compound* | **HL_5_** | **2** |
| --- | --- | --- |
| Formula | C_14_H_13_N_3_O | C_27_H_25_AuN_3_OP |
| M (g mol^-1^) | 239.27 | 635.44 |
| *T* (K) | 120(2) | 120(2) |
| Crystal system | Orthorhombic | Monoclinic |
| Space group | *P*bca | *P*2_1_/c |
| *a* (Å) | 7.5178(2) | 9.91540(17) |
| *b* (Å) | 23.5994(6) | 13.4387(2) |
| *c* (Å) | 27.4151(7) | 18.1518(3) |
| *α* (º) | 90 | 90 |
| *β* (º) | 90 | 91.3508(15) |
| *γ* (º) | 90 | 90 |
| *V* (A^3^) | 4863.9(2) | 2418.06(7) |
| *Z, D_c_* (g cm^-3^) | 16, 1.307 | 4, 1.745 |
| *F* (000) | 2016 | 1240 |
| *θ* range for data collection (º) | 2.94 ≤ *θ* ≤ 25.00 | 3.01 ≤ *θ* ≤ 25.00 |
| Reflections collected/unique | 43163 / 4275 | 21485 / 4248 |
| Data/restraints/parameters | 4275 / 0 / 325 | 4248 / 0 / 300 |
| Goodness-of-fit on *F^2^* | 1.075 | 1.027 |
| Final *R* indices [*I*>2σ(*I*)] | 0.0366, 0.0940 | 0.0174, 0.0435 |
| *R* indices (all data) | 0.0505, 0.0973 | 0.0197, 0.0440 |
| Largest peak and hole (e Å^-3^) | 0.465, -0.304 | 0.788, 0.416 |

**Table S2.** Selected bond lengths and angles (Å, °) for HL_5_. Data for two crystallographically independent molecules within the unit cell are presented in the following order: HL_5_/HL_5_A.

| O1–C6 | 1.346(2)/1.347(2) | N7–C8 | 1.361(2)/1.363(2) |
| --- | --- | --- | --- |
| O1–C10 | 1.446(2)/1.446(2) | C8–C9 | 1.366(2)/1.370(2) |
| N1–C6 | 1.321(2)/1.324(2) | C10–C11 | 1.502(2)/1.515(2) |
| N1–C2 | 1.352(2)/1.351(2) | C11–C12 | 1.516(2)/1.508(2) |
| C2–N3 | 1.318(2)/1.318(2) | C12–C13 | 1.377(2)/1.390(2) |
| N3–C4 | 1.369(2)/1.370(2) | C12–C17 | 1.386(2)/1.394 (2) |
| C5–N7 | 1.364(2)/1.364(2) | C13–C14 | 1.379(2)/1.389(2) |
| C4–C5 | 1.401(2)/1.406(2) | C14–C15 | 1.382(2)/1.380(2) |
| C4–C9 | 1.410(2)/1.406(2) | C15–C16 | 1.381(2)/1.379(2) |
| C5–C6 | 1.390(2)/1.388(2) | C16–C17 | 1.396(2)/1.381(2) |
|  |  |  |  |
| C6–O1–C10 | 118.61(11)/117.55(10) | C8–N7–C5 | 107.63(11)/107.60(11) |
| C6–N1–C2 | 116.89(12)/116.89(12) | N7–C8–C9 | 110.92(13)/110.78(13) |
| N3–C2–N1 | 128.54(13)/128.29(13) | C8–C9–C4 | 105.98(12)/106.19(12) |
| C2–N3–C4 | 114.21(12)/114.70(12) | O1–C10–C11 | 111.08(13)/113.48(12) |
| N3–C4–C5 | 121.35(13)/121.07(13) | C10–C11–C12 | 110.23(13)/114.47(12) |
| N3–C4–C9 | 131.52(12)/131.85(12) | C13–C12–C17 | 118.07(14)/117.92(14) |
| C5–C4–C9 | 107.12(12)/107.06(12) | C13–C12–C11 | 120.96(14)/120.91(13) |
| N7–C5–C6 | 133.21(12)/133.40(12) | C17–C12–C11 | 120.92(14)/121.17(13) |
| N7–C5–C4 | 108.34(12)/108.37(12) | C14–C13–C12 | 121.5(2)/121.02(14) |
| C6–C5–C4 | 118.42(12)/118.21(12) | C13–C14–C15 | 120.36(14)/119.9(2) |
| N1–C6–O1 | 121.81(12)/121.04(12) | C16–C15–C14 | 119.2(2)/119.8(2) |
| N1–C6–C5 | 120.54(12)/120.81(12) | C15–C16–C17 | 119.8 (2)/120.1(2) |
| O1–C6–C5 | 117.66(12)/ 118.15(12) | C12–C17–C16 | 121.00(14)/121.13(14) |

**Table S3.** Selected bond lengths and angles (Å, °) for complex **2**.

| Au1–N7 | 2.041(2) | N3–C4 | 1.366(3) |
| --- | --- | --- | --- |
| Au1–P1 | 2.2272(7) | C4–C5 | 1.407(4) |
| P1–C40 | 1.813(3) | C4–C9 | 1.415(4) |
| P1–C30 | 1.815(3) | C5–N7 | 1.367(3) |
| P1–C20 | 1.819(3) | C5–C6 | 1.394(4) |
| O1–C6 | 1.348(3) | N7–C8 | 1.358(3) |
| O1–C10 | 1.460(3) | C8–C9 | 1.362(4) |
| N1–C6 | 1.319(3) | C10–C11 | 1.492(5) |
| N1–C2 | 1.351(3) | C10–C12 | 1.505(4) |
| C2–N3 | 1.313(3) |  |  |
|  |  |  |  |
| N7–Au1–P1 | 176.35(6) | O1–C10–C12 | 105.8(2) |
| C40–P1–C30 | 104.63(12) | C11–C10–C12 | 113.3(3) |
| C40–P1–C20 | 104.94(12) | C5–C4–C9 | 105.9(2) |
| C30–P1–C20 | 107.36(12) | N7–C5–C6 | 132.9(2) |
| C40–P1–Au1 | 114.51(8) | N7–C5–C4 | 110.1(2) |
| C30–P1–Au1 | 110.04(8) | C6–C5–C4 | 117.0(2) |
| C20–P1–Au1 | 114.63(9) | N1–C6–O1 | 120.5(2) |
| C6–O1–C10 | 117.0(2) | N1–C6–C5 | 120.8(2) |
| C6–N1–C2 | 117.2(2) | O1–C6–C5 | 118.7(2) |
| N3–C2–N1 | 128.8(2) | C8–N7–C5 | 105.6(2) |
| C2–N3–C4 | 113.4(2) | C8–N7–Au1 | 129.1 (2) |
| N3–C4–C5 | 122.8(2) | C5–N7–Au1 | 124.6 (2) |
| N3–C4–C9 | 131.3(2) | N7–C8–C9 | 112.7(2) |
| C8–C9–C4 | 105.7(2) | O1–C10–C11 | 109.2(2) |

**Table S4.** Selected non-covalent contacts (Å, °) in the crystal structure of HL_5_.

| D–H...A | *d*(D–H) | *d*(H···A) | *d*(D···A) | <(DHA) |
| --- | --- | --- | --- | --- |
| N(7A)–H(7AA)···N(3) | 0.88 | 1.9850(12) | 2.8572(17) | 170.98(8) |
| N(7)–H(7A)···N(3A)^i^ | 0.88 | 1.9761(12) | 2.8430(12) | 168.27(8) |
| C(9)–H(9A)···O(1A) | 0.95 | 2.4999(9) | 3.3057(17) | 142.75(9) |
| C(9A)–H(9AA)···O(1)^i^ | 0.95 | 2.6646(10) | 3.3785(17) | 132.34(9) |
| C11–H(11C)···N3A^ii^ | 0.99 | 2.6829(11) | 3.313(2) | 121.70(9) |

Symmetry codes: (i) –x + 1/2, y – 1/2, z; (ii) x + 1/2, –y + 1/2, –z + 1.

**Table S5.** Selected non-covalent contacts (Å, °) in the crystal structure of **2**.

| D–H...A | *d*(D–H) | *d*(H···A) | *d*(D···A) | <(DHA) |
| --- | --- | --- | --- | --- |
| C12–H12B···C45^i^ | 0.980(4) | 2.851(3) | 3.465(4) | 121.5(2) |
| C41–H41···C4^ii^ | 0.950(3) | 2.679(2) | 3.568(4) | 156.1(2) |
| C41–H41···N3^ii^ | 0.950(3) | 2.620(2) | 3.282(4) | 127.1(2) |
| C45–H45A···C8^iii^ | 0.950(3) | 2.803(3) | 3.509(4) | 131.8(2) |
| C44–H44A···Cg^iii^ | 0.950(3) | 2.7298 | 3.525(3) | 141.6(2) |
| C34–H34A···N3^iv^ | 0.95(3) | 2.580(2) | 3.433(4) | 149.6(2) |
| C33–H33A···N1^v^ | 0.949(3) | 2.717(2) | 3.523(4) | 143.2(2) |

Symmetry codes: (i) – x, y – 1/2, –z + 1/2; (ii) –1 + x, –y, –z + 1; (iii) – x, –y,–z + 1; (iv) x, –y – 1/2, z – 1/2; (v) –x + 1, y + 1/2, –z + 1/2.


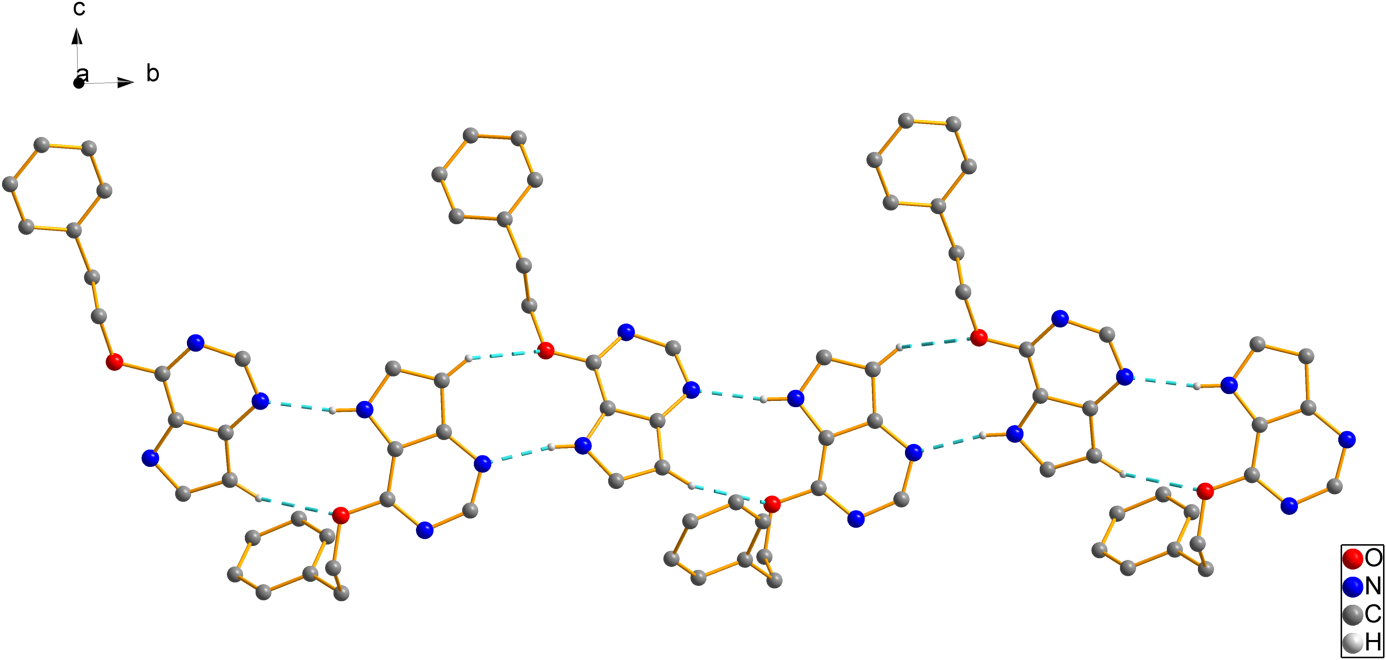


**Figure S5.** A part of the crystal structure of HL_5_, showing the N–H∙∙∙N and C–H∙∙∙O hydrogen bonds (dashed lines), and the formation of one-dimensional supramolecular chain. The hydrogen atoms not involved in the depicted contacts were omitted for clarity.
